# A Cbx8-Containing Polycomb Complex Facilitates the Transition to Gene Activation during ES Cell Differentiation

**DOI:** 10.1371/journal.pgen.1004851

**Published:** 2014-12-11

**Authors:** Catherine Creppe, Anna Palau, Roberto Malinverni, Vanesa Valero, Marcus Buschbeck

**Affiliations:** Institute of Predictive and Personalized Medicine of Cancer (IMPPC), Badalona, Barcelona, Spain; University of Oslo, Norway

## Abstract

Polycomb proteins play an essential role in maintaining the repression of developmental genes in self-renewing embryonic stem cells. The exact mechanism allowing the derepression of polycomb target genes during cell differentiation remains unclear. Our project aimed to identify Cbx8 binding sites in differentiating mouse embryonic stem cells. Therefore, we used a genome-wide chromatin immunoprecipitation of endogenous Cbx8 coupled to direct massive parallel sequencing (ChIP-Seq). Our analysis identified 171 high confidence peaks. By crossing our data with previously published microarray analysis, we show that several differentiation genes transiently recruit Cbx8 during their early activation. Depletion of Cbx8 partially impairs the transcriptional activation of these genes. Both interaction analysis, as well as chromatin immunoprecipitation experiments support the idea that activating Cbx8 acts in the context of an intact PRC1 complex. Prolonged gene activation results in eviction of PRC1 despite persisting H3K27me3 and H2A ubiquitination. The composition of PRC1 is highly modular and changes when embryonic stem cells commit to differentiation. We further demonstrate that the exchange of Cbx7 for Cbx8 is required for the effective activation of differentiation genes. Taken together, our results establish a function for a Cbx8-containing complex in facilitating the transition from a Polycomb-repressed chromatin state to an active state. As this affects several key regulatory differentiation genes this mechanism is likely to contribute to the robust execution of differentiation programs.

## Author Summary

Cell fate transitions have long been known to be accompanied by alterations in chromatin structure. But only during the last few years has it become clear that chromatin modifications form the molecular basis of an epigenetic memory that defines cell identity. The Polycomb Group Proteins (PcGs) form two major protein complexes known as polycomb repressive complexes 1 and 2 (PRC1 and PRC2). Their function is essential for the maintenance of transcriptional repression during embryogenesis through the methylation of the lysine 27 on histone H3 and the subsequent ubiquitination of histone H2A. The chromobox homolog 8, Cbx8, which is part of the PRC1 complex, is therefore generally defined as a repressor of gene transcription. The genome wide profiling of Cbx8 during the early steps of mouse embryonic stem (mES) cells differentiation provided us with surprising results involving Cbx8 in gene activation. Our results point out that Cbx8 is part of a PRC1 complex involved in the transition from a Polycomb repressed state to an active state.

## Introduction

First identified in *Drosophila*, the polycomb group of proteins share conserved domains and play an important role in coordinated gene repression during vertebrate and invertebrate development [1]. The prevailing view is that PRC2 and PRC1 act in a sequential manner. The association of the three proteins Eed, Suz12 and Ezh2 or Ezh1 leads to the formation of the core PRC2 complex. Ezh1 and Ezh2 are histone methyl transferases that mediate the addition of up to three methyl groups to lysine 27 of histone H3 (H3K27me1–3). The trimethylated mark is recognized by PRC1 complexes that further mediate ubiquitination of H2A and gene repression [2]–[4]. More recently, it has been shown that PRC1 can also be recruited to chromatin in the absence of a functional PRC2 complex [5]–[8].

In contrast to PRC2, the composition of PRC1 is highly modular and much more variable. The ubiquitin ligase that provides the catalytic activity to the PRC1 complex can be either Ring1a or Ring1b. The complex additionally includes one of six Pcgf proteins; one of three orthologs of polyhomeiotic and five mutually exclusive Cbx proteins can occupy the position of the *Drosophila* Polycomb protein. Cbx proteins differ in some of their domains suggesting that they could convey different functional and regulatory properties to PRC1 [9]. In addition a variant complex in which RYBP replaces Cbx proteins has been shown to mediate repression independent of the methylation status of H3K27 [7].

Mouse embryonic stem (ES) cells are characterized by their ability to self-renew and their potential to differentiate into any of the three germ layers. PRC maintain the pluripotency of the cells by maintaining the developmental regulators repressed [10]–[12]. On differentiation ES cells acquire cell-type specific gene expression patterns that strongly depend on the genome-wide redistribution of the Polycomb proteins [12].

Activation of tissue specific genes correlates with the displacement of Polycomb proteins and a decrease of the H3K27me3 mark during retinoic acid induced neuronal differentiation [13]. However, it has been recently shown that Polycomb proteins can also be recruited to activated genes to attenuate the retinoic acid associated transcriptional activation of specific genes [14]. The important function of Polycomb complexes in the epigenetic changes induced by retinoic acid in mouse embryonic stem cells has been recently reviewed by Gudas [15].

The composition of the PRC1 complex changes during the differentiation of ES cells. Cbx7 is the primarily expressed Polycomb ortholog in ES cells but it is quickly downregulated during differentiation while Cbx2, Cbx4 and Cbx8 are induced [16], [17]. These studies showed that the integrity of Cbx7 was required for stable ES cell maintenance, while Cbx2 and Cbx4 were required for balanced lineage specification. It is worth noting that similar results have been obtained for hematopoietic stem cells [18].

However, some important questions about Polycomb proteins remain unanswered. Despite their overt relevance for ES cell differentiation, it is poorly understood how Polycomb repressed states are established and resolved. How PRCs are initially recruited to target genes is a matter of continuous debate (discussed in [1]). Similarly it is unclear how the transition from a PRC repressed state to an active state is achieved. How changes in PRC composition relate to these transitions has not been investigated. Here, we analyzed the genome wide recruitment of Cbx8 in ES cells induced to differentiate. We provide compelling evidence suggesting that Cbx8 is part of a transitory PRC1 complex facilitating the activation of Cbx7-PRC1-repressed genes during the commitment to differentiation.

## Results

### During ES cell differentiation Cbx8 is recruited to activated developmental genes

**Figure 1:**
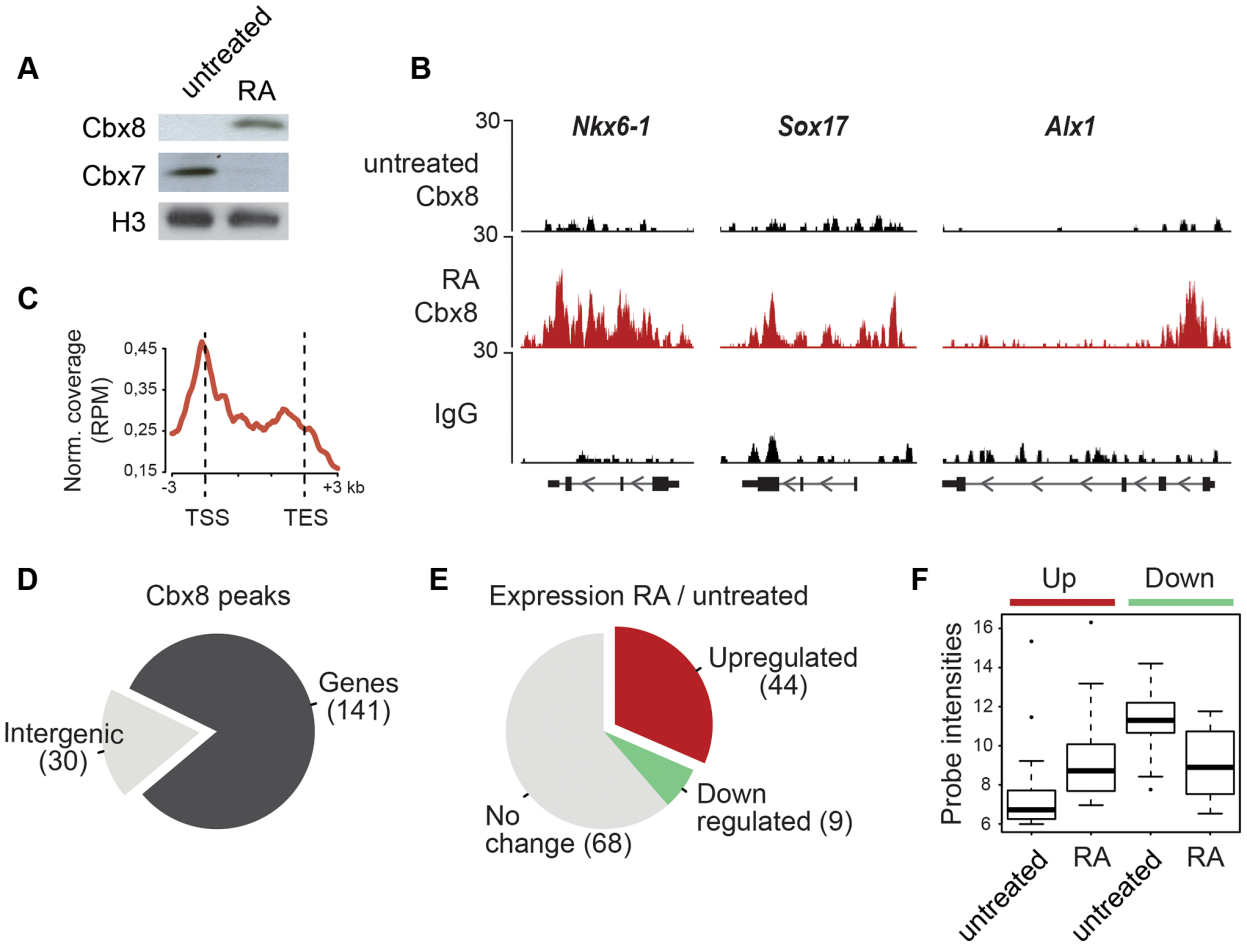
Cbx8 is upregulated and recruited to activated genes during ES cell differentiation. (A) Lysates from E14 mouse embryonic stem (mES) cells treated or not with 1 µM retinoic acid (RA) during 3 days were analyzed by Western blotting using anti-Cbx8 and anti-Cbx7 antibodies. Histone H3 was used as loading control. (B) ChIP-seq profiles of IgG and Cbx8 of selected genes in ES cells treated with RA for 3 days or left untreated are shown using the UCSC Genome Browser. (C) Normalized read distribution of genes with associated Cbx8 peaks in ES cells treated with RA for three days. Please note that average read distribution on gene bodies is plotted after normalization for the size of transcribed regions, while regions upstream of the transcription start site (TSS) and downstream of the transcription end site (TES) are plotted linear. (D) The large majority of Cbx8 peaks are associated with genes considering a window of the transcribed region ±3 kb. (E) For 121 of the 141 gene-associated peaks we extracted transcriptional information from whole genome expression microarrays of ES cells treated with RA for 3 days and untreated control cells. The proportion of genes with upregulated, downregulated or unchanged expression using a 1.5-fold change as cut-off is plotted in a pie chart and absolute numbers are indicated in brackets. (F) Average probe intensities corresponding to the up- and downregulated Cbx8-target genes indicated in E are shown in a boxplot for untreated and RA-treated cells. Note that probe intensities from 6-7 correspond to background levels. All data plotted in D–F is detailed in S1 Table.

**Figure 2:**
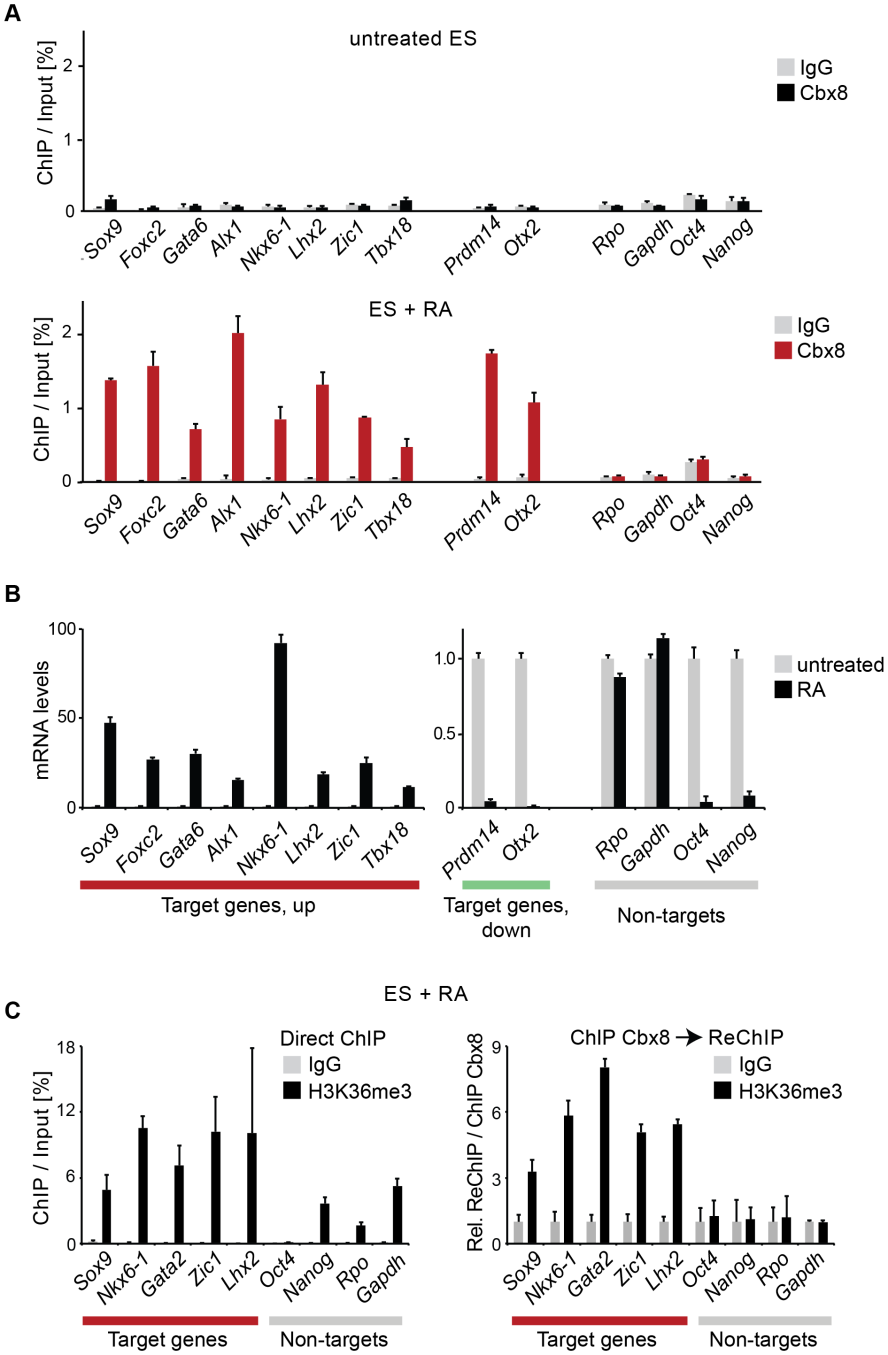
Cbx8 binding coincides with gene activation. (A) The occupancy of Cbx8 on genes was analyzed by ChIP in untreated ES cells and differentiating cells treated with RA for 3 days. Data is plotted as percentage of ChIP-enriched DNA in respect to DNA from input material. Error bars denote s.d., and n = 3. (B) qRT-PCR analysis of mRNA levels in the same cells as A. Levels in untreated cells have been set to one. Error bars denote s.d., and n = 3. (C) ChIP and ReChIP analyses performed with differentiating ES cells treated with RA for three days are shown. Anti-H3K36me3 and control IgG antibody were used in a primary ChIP (left panel) or in a secondary ChIP performed on material enriched in a primary ChIP with anti-Cbx8 antibody (right panel). ReChIP data is plotted relative to IgG. Error bars denote s.d., n = 3.

We used retinoic acid (RA) to induce mouse E14 ES cells to start differentiating towards the neuronal lineage. We confirmed previous results [16], [17] showing that Cbx8 was virtually absent in self-renewing ES cells but potently induced on protein and RNA levels after three days of RA-induced differentiation (Fig. 1A and S1A Figure). To assess the genome wide distribution of Cbx8 in differentiating ES cells, we enriched Cbx8-bound chromatin by chromatin immunoprecipitation (ChIP) using an antibody generated against the unique part of the protein (S1B-G Figure) and analyzed the co-precipitated DNA by direct massive parallel sequencing (ChIP-seq). Taking advantage of the fact that Cbx8 is virtually absent in untreated, self-renewing ES cells (Fig. 1A), we decided to use both IgG as well as Cbx8 ChIPs from untreated cells as negative controls. We were able to uniquely map 9–16 million reads per sample (S2A Figure). For our further analysis we used a set of high confidence binding sites that were identified by the overlap of peaks that were called by MACS comparing Cbx8 ChIP from RA-treated ES cells to IgG and those called comparing Cbx8 ChIP from RA-treated ES cells to the antibody-specific background ChIPed from untreated cells (S2B Figure). By this method we were able to identify a subset of 171 peaks corresponding to Cbx8 binding sites of high confidence (S1 Table). Peaks were annotated to the nearest gene if the center of the peak was inside a window flanking the transcribed region by 3 kb. Plotting the average read coverage on genes with annotated Cbx8 indicated that Cbx8 tends to accumulate on gene bodies but also to spread into upstream and downstream regions (Fig. 1C). Using this annotation we found that the large majority (141/171) of identified peaks are associated to annotated genes (Fig. 1D). We performed a microarray analysis comparing self renewing untreated ES cells and differentiating cells after 3 days of RA treatment and crossed the data with our ChIP-seq to identify possible transcriptional changes on Cbx8 target genes. Taking into consideration the established function of Polycomb proteins in gene repression, we expected target genes to either not change because they are maintained in a repressed state or to be downregulated. We could extract data for 121 of the 141 gene-associated peaks. We found that about one third of these Cbx8 binding sites (53 peaks) annotated to genes that displayed a more than 1.5-fold change in gene expression. To our surprise the large majority of these genes (44/53) was not down- but upregulated (Fig. 1E). Most of these upregulated genes were repressed in untreated cells as indicated by very low average probe intensities (Fig. 1F). Though a previous report has already shown that Cbx8 can be found on a handful of activated genes in differentiating cells [19], our data suggested that this could actually be true for a substantial fraction of Cbx8 target genes. In order to confirm this, we selected a panel of target and control genes and simultaneously analyzed Cbx8 recruitment and the corresponding mRNA levels. We were able to confirm differentiation-induced recruitment of Cbx8 on all target genes tested while control genes were negative (Fig. 2A). Target genes included many important key differentiation genes such as *Sox9*, *Gata6* and *Nkx6-1* whose expression was potently induced (Fig. 2B). In order to exclude the possibility that Cbx8 binding and active transcription might occur on different and exclusive alleles within the cell population, we analyzed the co-occurrence of Cbx8 and H3K36me3, which is a mark of active transcription [20], by coupled ChIP in differentiating ES cells treated for three days with RA. First, we have analyzed five Cbx8 target genes and four non-target genes that included *Oct4*, *Nanog*, *Gapdh* and *Rpo*. Despite the fact that *Nanog* is downregulated after three days of RA treatment (Fig. 2B) it still retained some H3K36me3 that was in a similar range as on the activated gene *Sox9* or the constitutively active gene *Gapdh* (Fig. 2C, left panel) suggesting that removal of the active mark H3K36me3 follows a slower dynamic than the actual gene repression. Taking advantage of the fact that with *Gapdh*, *Nanog*, and *Sox9* we had identified target and non-target genes of Cbx8 with comparable H3K36me3 levels, we have used anti-Cbx8 antibody-enriched chromatin as input material for a secondary ChIP with IgG and anti-H3K36me3 antibody. As shown in the right panel of Fig. 2C, we found a clear enrichment of H3K36me3 over IgG on *Sox9* and on the other Cbx8 target genes but not on non-target genes such as *Gapdh* or *Nanog*. As chromatin ranging in size between 300–500 bps has been used for these experiments H3K36me3 and binding of Cbx8 could be occurring on different H3 tails on the same or even neighboring nucleosomes. The observation that Cbx8 can be simultaneously detected on the same locus provides further support to the idea that Cbx8 is recruited to genes that become actively transcribed.

### Cbx8 facilitates gene activation

**Figure 3:**
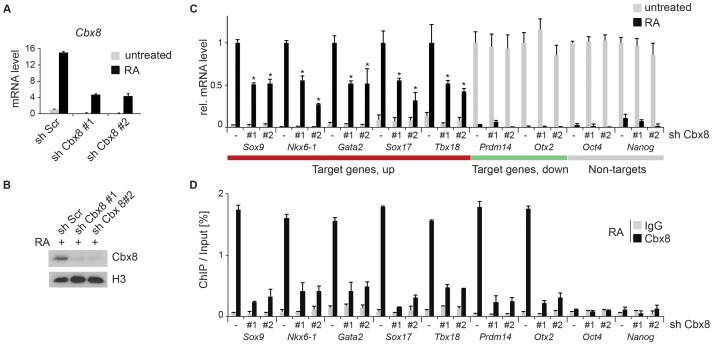
Knockdown of Cbx8 reduces transcription of activated target genes. (A) The mRNA levels of Cbx8 in control cells (sh Scr) and after shRNA-mediated knockdown of Cbx8 using lentiviral transduction of two independent hairpins (#1 and #2) were analyzed by qRT-PCR before and after treatment with RA. Error bars denote s.d., and n = 3. (B) Western blot analysis of Cbx8 in RA-treated ES cells. Histone H3 was used as loading control. (C) Transcript expression levels of genes in cells expressing Cbx8-specific shRNAs or control shRNA (indicated by minus) were analyzed. Color code of target genes is according to Figs. 1F and 2B. For each gene, values are plotted relatively to control treated with RA. Error bars denote s.d.; n = 3; *, p<0.05 (comparing to RA-treated control). (D) The occupancy of Cbx8 on the same genes was analyzed by ChIP. Error bars denote s.d., and n = 3.

**Figure 4:**
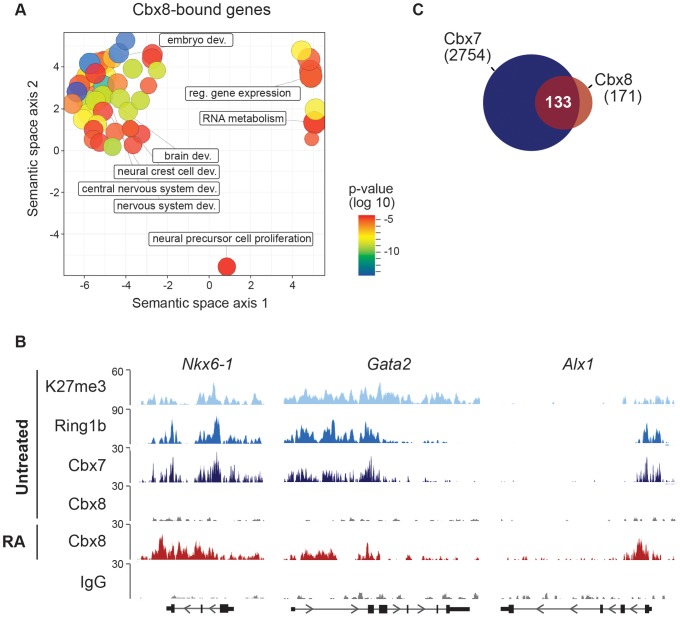
Cbx8 target genes are PRC1 target genes in self-renewing ES cells. (A) Cbx8-enriched GO categories are visualized using REVIGO [48] which allows to cluster GO according to their similarity in a semantic space. Only GO categories with an adjusted P-value of 0.001 or less are shown using “medium” for the allowed semantic similarity. (B) ChIP-seq profiles of H3K27me3, Ring1b, Cbx7 and Cbx8 in ES cells in self-renewing conditions and of Cbx8 and IgG in RA-treated ES cells are shown using the UCSC genome browser. (C) Venn diagram showing the overlap of Cbx8 target genes in RA-treated cells with those of Cbx7 in untreated, self-renewing ES cells.

To study the functional importance of Cbx8 for the activation of differentiation genes, we used lentiviral vectors expressing two different short hairpin RNAs (shRNA) directed specifically against the Cbx8 transcript. After selection stably transduced cells were used for the study. Both shRNAs efficiently repressed the expression of Cbx8 on both the mRNA and protein levels in ES cells treated with RA (Fig. 3A,B). The activation of upregulated Cbx8 target genes was significantly decreased in cells depleted for Cbx8 (Fig. 3C). Among the Cbx8-sensitive genes were several pivotal regulators of differentiation processes such as *Sox9* and *Nkx6-1*, that have been shown to be important transcription factors required for normal brain development [21], [22]. The reduction of Cbx8 occupancy was similarly efficient on up- as well as downregulated genes (Fig. 3D), however, the reduction in Cbx8 levels didn't affect the repression of its target genes *Prdm14* and *Otx2* (Fig. 3C). Importantly, the non-target genes *Oct4* and *Nanog*, which encode the regulators of pluripotency, were similarly repressed in Cbx8 deficient and control cells (Fig. 3C,D). Plotting enriched gene ontologies according to their similarity in a semantic space illustrates a clear overrepresentation of both transcriptional and differentiation regulators (Fig. 4A), which are the classical categories of Polycomb target genes in self-renewing ES cells [12]. Gene ontologies related to neuronal development were preferentially enriched in the subgroup of activated Cbx8 genes but not those target genes that did not show any change in gene expression (S3A Figure). Downregulated genes were not sufficient in number to yield a result in gene ontology analysis. We compared our genome wide Cbx8 binding profile in differentiating ES cells with published ChIP-seq data obtained from self-renewing ES cells [17]. The binding of Cbx8 in RA-treated differentiating ES cells mirrors the binding of PRC1 proteins Ring1b and Cbx7 within H3K27me3 domains in untreated self-renewing ES cells (Fig. 4B). As shown in Fig. 4C, this holds true for the vast majority of Cbx8 binding sites in RA-treated ES cells as 133/171 overlapped with sites bound by Cbx7 in self-renewing ES cells. Similar overlaps were observed with Ring1b and H3K27me3 (S3B Figure).

### Cbx8 acts as part of a PRC1 complex

**Figure 5:**
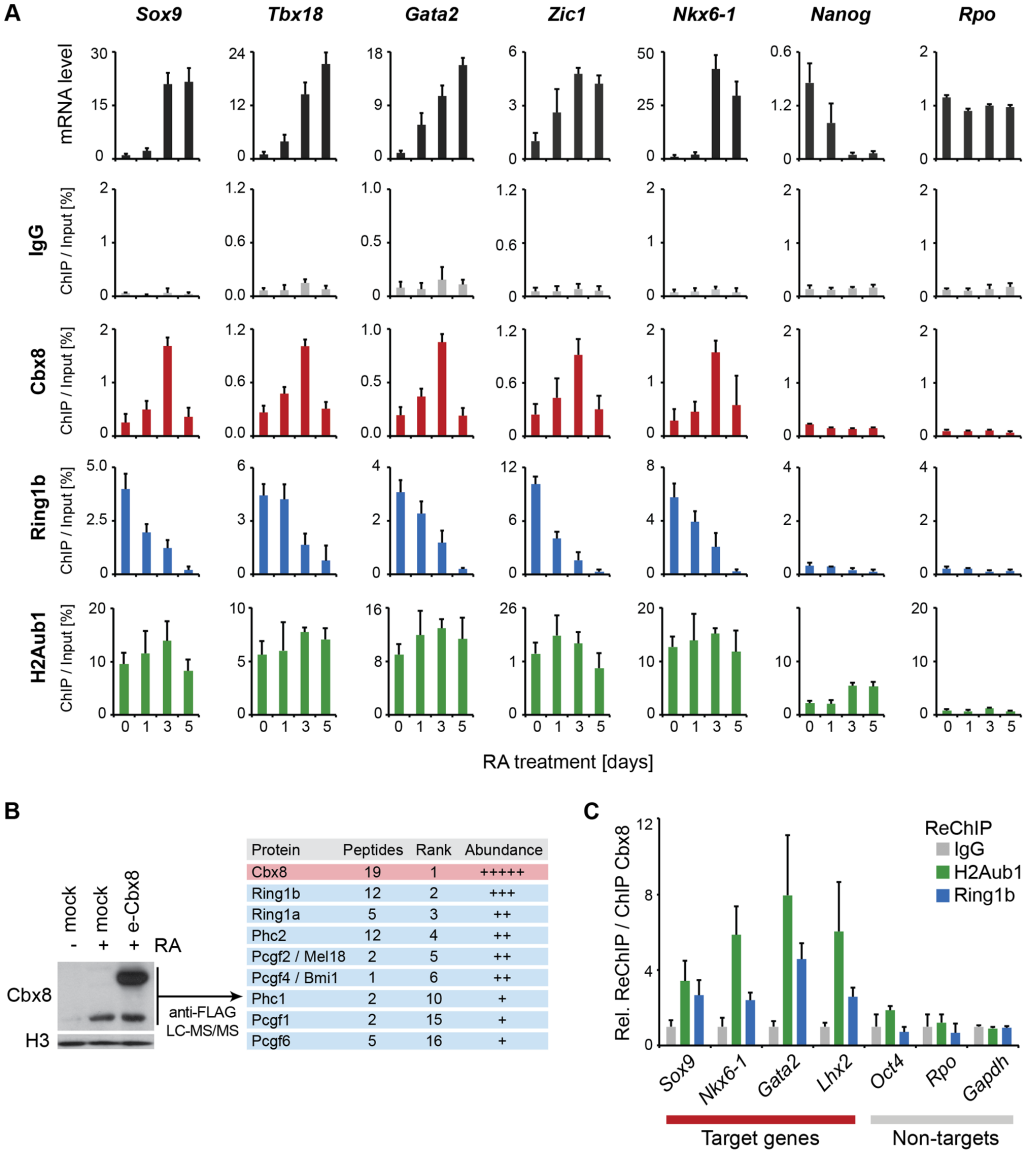
Cbx8 is part of a PRC1 complex and transiently bound to activated genes. (A) Time course of RA-induced differentiation of ES cells. mRNA levels and occupancy by Cbx8, Ring1b and H2A K117 mono-ubiquitination (H2Aub1) were analyzed by qRT-PCR and ChIP, respectively. Error bars denote s.d.; n≥3. (B) FLAG-affinity purifications from mock and FLAG epitope-tagged (e)-Cbx8 expressing cells treated with RA for 3 days, analyzed by LC-MS/MS. Enriched proteins were ranked according to abundance (see also S2 Table). Relative abundance to bait is indicated with +, 1–10%; ++, 10–30%; +++, 30–50%; and ++++, >50%. (C) ReChIPs using anti-Ring1b, anti-H2Aub1 antibodies and IgG as control were performed on material enriched in a primary ChIP with anti-Cbx8 antibody. Data is plotted relative to IgG. Error bars denote the variation of the mean of two independent experiments.

The unexpected link between Cbx8 recruitment and gene activation prompted us to analyze whether Cbx8 acted alone, as part of a PRC1 or part of a different complex. First we compared the dynamics in occupancy of Cbx8 and Ring1b, which is the least variant component of PRC1. As already suggested by the ChIP-seq data (Fig. 4B), Ring1b was strongly enriched on target genes in self-renewing ES cells. While the recruitment of Cbx8 initially increased and peaked after three days of retinoic acid induction, the binding of Ring1b decreased progressively reaching background levels at day five of differentiation (Fig. 5A). At day five of retinoic acid induced differentiation, the Cbx8 occupancy dropped to very low levels similar to Ring1b. In contrast, H2A ubiquitination, mark set by Ring1b [23], persisted over the entire time course (Fig. 5A). In order to understand whether Cbx8 and Ring1b are acting together, we decided to identify the proteins that bind Cbx8 during ES cell differentiation. Therefore, we generated ES cells stably expressing epitope-tagged Cbx8 (Fig. 5B). Exogenous Cbx8 expressed in untreated ES cells bound to the same target genes as endogenous Cbx8 in RA-treated ES cells suggesting that the epitope does not affect its function (S4A Figure). We harvested cells at day three of differentiation which corresponds to the time point with maximal recruitment of endogenous Cbx8 to target genes (Fig. 5A). Cbx8 and interacting proteins were enriched by affinity purification, analyzed by mass spectrometry and significantly enriched proteins were ranked according to their abundance (S2 Table). After the bait protein Cbx8, the top five ranked proteins were the PRC1 subunits Ring1a/b, Phc2 and the Pcgf proteins Mel18 and Bmi1. Three additional PRC1 subunits were found in lower abundance (Fig.5B). Next, we tested whether Ring1b and H2A ubiquitination co-occur with Cbx8 on genes. Therefore, we have used anti-Cbx8 antibody-enriched chromatin as input material for a secondary ChIP with IgG, anti-Ring1b and anti-ubiquitinated H2A antibody. As shown in Fig. 5C we could detect co-enrichment on the target genes *Sox9*, *Nkx6-1*, *Lhx2* and *Gata2* but not on the non-target genes *Oct4*, *Rpo* or *Gapdh*. Taken together, these results suggested that Cbx8 acts as part of a PRC1 complex.

**Figure 6:**
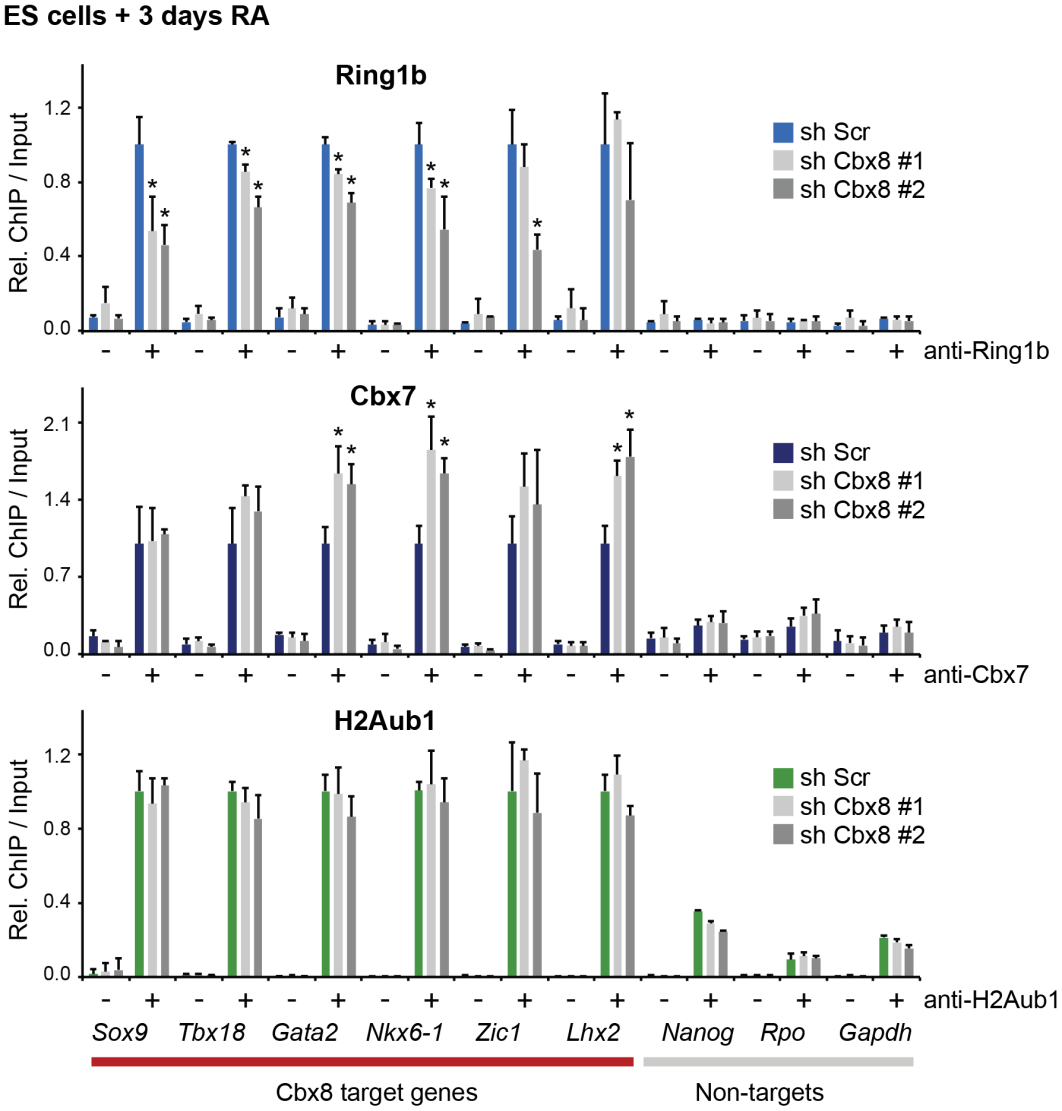
Loss of Cbx8 is partially compensated by Cbx7. The presence of Ring1b, Cbx7 and H2A ubiquitination (H2Aub1) in control cells (sh Scr) and after shRNA-mediated knockdown of Cbx8 using two independent hairpins was analyzed by ChIP after 3 days RA treatment. IgG was used as background control and is indicated by minus signs. For each Cbx8 target gene values are plotted relatively to control cells. Enrichments on-target genes are plotted relative to the average enrichment on all six target genes in control cells. Error bars denote s.d.; n = 3; *, p<0.05. (compared to control cells).

To further support this, we have analyzed the occupancy of Cbx8 target genes by Ring1b, Cbx7 and H2A ubiquitination in RA-treated cells after Cbx8 knockdown. The resulting reduction of Cbx8 occupancy (Fig. 3D) correlated with a small but consistent reduction of Ring1b on several Cbx8 target genes without affecting non-target genes (Fig. 6). Notably, global Ring1b protein levels were not affected (S4B Figure). However, we observed an increased incorporation of Cbx7 that could partially compensate for Cbx8 loss (Fig. 6). This was not the consequence of an upregulation of Cbx7 expression as knockdown of Cbx8 did not affect the mRNA levels of Cbx7 or other Cbx proteins (S4C Figure). Moreover we found that Cbx8 loss did not affect the levels of H2A ubiquitination on its target genes (Fig. 6). In order to address the question whether Cbx8-containing PRC1 on activated genes is repressive or activating, we stably interfered with the expression of Ring1b, the least variant component of PRC1. When analyzing Ring1b knockdown cell after 3 days of RA treatment we did not observe any compensation by Ring1a but a slight increase in Cbx8 mRNA (S4D Figure). Under these conditions the Cbx8 target gene *Gata2* was further upregulated while other target genes such as Nkx6-1 and Sox17 were less activated (S4D Figure). These results are difficult to interpret as knockdown of Ring1b interferes with all PRC1 complexes present prior and after RA treatment.

### Cbx8-containing PRC1 binds to persisting H3K27me3

**Figure 7:**
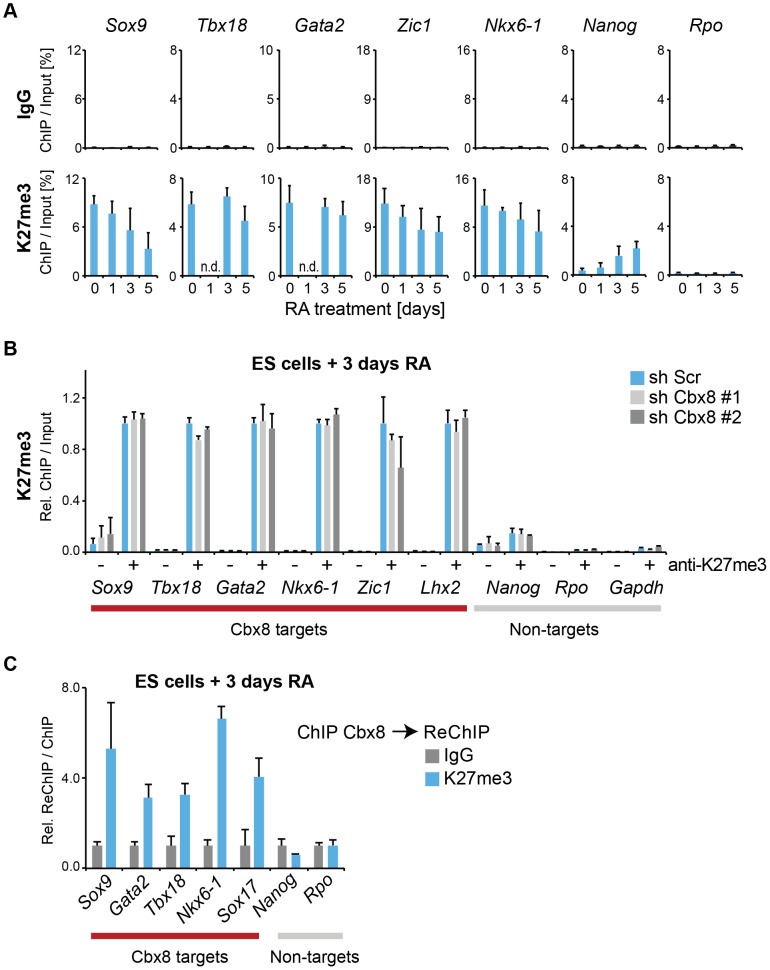
Cbx8 binds to persisting K27 methylation during differentiation. (A) Trimethylation of lysine 27 of Histone H3 was analyzed by ChIP during a time course of RA-induced differentiation of ES cells. Control ChIPs with IgG are shown in the top panel on the same scale. Error bars denote s.d.; n≥3; n.d.  =  not done. (B) ChIP of H3K27me3 (+) and IgG (-). For each Cbx8 target gene values are plotted relatively to control cells. Enrichments on-target genes are plotted relative to the average enrichment on all six target genes in control cells. Error bars denote s.d.; n = 3. (C) ReChIPs using anti-K27me3 antibody and IgG as control were performed on material enriched in a primary ChIP with anti-Cbx8 antibody. Data is plotted relative to IgG. Error bars denote the variation of the mean of two independent experiments.

We then focused our attention on the mechanism that recruits Cbx8 to its target genes. An obvious possibility is that it binds directly to H3K27me3. Although activated Cbx8 target genes progressively reduced their H3K27me3 levels, in contrast to Ring1b, this reduction occurred only very slowly and even after five days of differentiation genes still retained half-maximal or even higher levels of H3K27me3 (Fig. 7A). Depletion of Cbx8 did not affect the amount of H3K27me3 detectable 3 days after RA treatment (Fig. 7B). We then analyzed by ChIP-ReChIP if Cbx8 and H3K27me3 co-occur on target genes in ES cells treated for 3 days with RA. Indeed, we found that Cbx8 and H3K27me3 co-existed on Cbx8 target genes (Fig. 7C). This suggests that also in the context of gene activation the interaction of Cbx8 with H3K27me3 is the most likely mechanism of recruitment to chromatin.

In addition to H3K27me3, at day 3 of RA treatment we also detected some acetylation of the same residue on Cbx8 target genes (S5B Figure). This H3K27ac was of low level when compared to an enhancer that was previously described to be marked by H3K27ac in ES cells (S5A Figure) [24]. Curiously, knockdown of Cbx8 lead to a modest but significant decrease of H3K27ac (S5B Figure). The presence of these two mutually exclusive H3K27 marks provided us with a valuable tool to interrogate the preferential binding of Cbx8. First we titrated both antibodies to reach similar enrichment in ChIP assays (S5C Figure). Then we used the enriched material to perform a sequential ChIP for Cbx8. As shown in S5C Figure, Cbx8 preferentially bound K27me3-marked chromatin.

### Cbx8 is required for efficient gene activation but not sufficient to induce it

**Figure 8:**
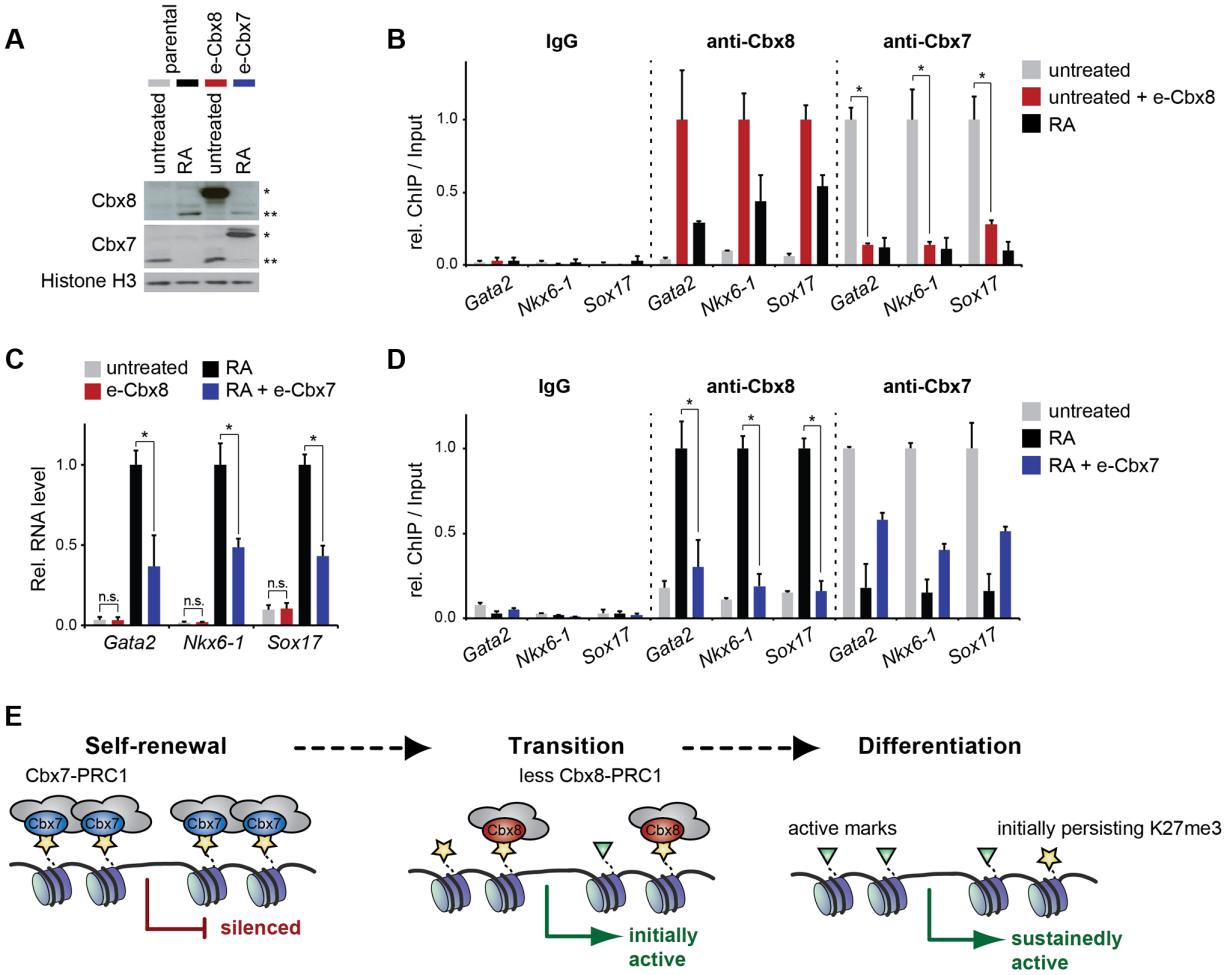
The exchange of Cbx7 for Cbx8 is required but not sufficient to promote the differentiation of mES cells. (A-D) Parental ES cells treated with RA for three days or left untreated were compared to untreated ES cells expressing exogenous Cbx8 and differentiating ES treated with RA for three days and expressing exogenous Cbx7. Consistent color coding of cells and treatments is as indicated in A. (A) The expression levels of endogenous and exogenous epitope (e)-tagged Cbx proteins is shown by Western blot of lysates from stable transfected ES cells (e-Cbx7 and e-Cbx8, respectively) and parental control cells. Exogenous and endogenous protein bands are marked by one or two asterisks, respectively. Cells were treated with RA for three days as indicated. Histone H3 was used as loading control. (B) The occupancy of Cbx7 and Cbx8 on selected genes was analyzed by ChIP in untreated ES cells overexpressing Cbx8 (red bars) and parental control cells treated with RA for three days (black bars) or left untreated (grey bars). ChIP-Input ratios for each gene and antibody are shown relative to the maximal enrichment. IgG is shown relative to Cbx8 ChIP. Error bars denote s.d.; n = 3; * = p<0.05. (C) Relative mRNA levels of the same genes as in B measured by qRT-PCR are shown. Error bars denote s.d.; n = 3; * = p<0.05; n.s.  =  not significant. (D) ChIP of Cbx8 or Cbx7 in Cbx7-overexpressing cells treated with RA for three days (blue bars) and control cells treated with RA (black bars) or left untreated (grey bars). ChIP-Input ratios for each gene and antibody are shown relative to the maximal enrichment. IgG is shown relative to Cbx8 ChIP. Error bars denote s.d.; n = 3; * = p<0.05. (E) Cartoon illustrating our finding that Cbx8-containing PRC1 complexes replace Cbx7-containing PRC1 complexes during the initial activation of differentiation genes. During a metastable transition state binding of Cbx8-containing PRC1 co-occurs with the presence of both repressive marks such as H3K27me3 and active marks such as H3K36me3. Prolonged activation results in loss of Cbx8-containing PRC1 that precedes the removal of H3K27me3.

Cbx7 and Cbx8 are expressed in an almost mutually exclusive manner in self-renewing and differentiating ES cells, respectively [16], [17]. To further gain additional insight into the functional relevance of the switch from Cbx7 to Cbx8 on target genes, we generated mouse embryonic stem cells that stably express exogenous Cbx8 and analyzed them in self-renewing conditions while cells expressing exogenous Cbx7 were analyzed in differentiating cells after 3 days of retinoic acid induction (Fig. 8A). When expressed in self-renewing cells, exogenous Cbx8 was able to efficiently outcompete Cbx7 for its target genes (Fig. 8B). The enforced recruitment of exogenous Cbx8 achieved under these conditions was two-to-three-fold higher than that observed in differentiating cells for the endogenous protein (Fig. 8B), although, this did not affect the low expression level of these genes (Fig. 8C). In the converse experiment during differentiation, Cbx7 overexpression significantly reduced the activation of Cbx8 target genes (Fig. 8C). Although enrichment of overexpressed Cbx7 on target genes did not reach the levels of the endogenous protein in self-renewing cells, it resulted in an efficient displacement of Cbx8 (Fig. 8D).

## Discussion

### Cbx8 is part of a transition PRC1 acting during initial gene activation

Taken together our results support a model in which PRC1 containing Cbx8 replace PRC1 containing Cbx7 on developmental genes, which facilitates the transition from a repressed chromatin state to gene activation during early ES cell differentiation (Fig. 8E). Prolonged gene activation results in eviction of PRC1 complexes despite some persisting H3K27me3 and H2A ubiquitination. This mechanism affects several key regulatory genes and thus probably contributes to a robust execution of differentiation programs.

Our data supports the idea that Cbx8 acts in the context of an intact PRC1 complex. Initially, we considered also two other possibilities: Cbx8 could act as monomer competing with repressive PRC1 complexes for H3K27me3 binding sites, or Cbx8 acts in complex with non-polycomb proteins that are activating. Indeed, in leukemia cells Cbx8 has already been described as a component of activating complexes containing Tip60 and MLL-AF9 [25]. However, performing mass spectrometric analysis of affinity purified Cbx8-complexes from differentiating ES cells we could not detect any of these proteins, but rather found other PRC1-components acting as main binding proteins (Fig. 5B). Moreover, we have shown that Ring1b was enriched on Cbx8 immunoprecipitated chromatin compared to IgG (Fig. 5C) and that the knockdown of Cbx8 resulted in a small but detectable reduction of Ring1b occupancy on genes (Fig. 6). Since we would have expected the opposite if Cbx8 acted as a monomer on target genes, this observation taken together with our co-immunoprecipitation data strongly argued for Cbx8 to be functioning in the context of a PRC1 complex.

### Replacement of Cbx7-containing PRC1 for a Cbx8-containing complex

One fundamental question is how a Cbx8-containing PRC1 is able to contribute to gene activation. A plausible explanation could be that Cbx8-containing PRC1 is simply less repressive than the PRC1 containing Cbx7 that it replaces. This prompted us to test whether Cbx8 has an influence on PRC1 activity. When monitoring the ubiquitination of H2A, we found that its levels on Cbx8 target genes neither decreased during the first five days of differentiation nor changed on depletion of Cbx8 (Figs. 5A, 6). It has been shown that Ring1b mediates chromatin compaction and gene repression independently of its catalytic activity [26]. In that regard, it would be interesting to test whether changes in PRC1 composition affects its capacity to compact chromatin. If Cbx7-containing PRC1 complexes induced a higher degree of chromatin compaction, this could possibly be relaxed on replacement by Cbx8. Knockdown of Ring1b itself let to the further activation of a Cbx8 target gene but to a reduction in the activation of several other Cbx8 target (S4D Figure). It is intriguing to speculate that the outcome could depend on the ratio of Cbx7 and Cbx8-containing PRC1 complexes present at the time point of analysis and the gene-specific kinetics of activation. However, the interpretation of such data is complicated by the large number of binding sites of Ring1b as its other target genes could exert indirect effects and the fact that Ring1b occurs in both PRC1 and non-PRC1 complexes [27]. Finally, of course we cannot exclude the possibility that Cbx8-containing PRC1 complexes are able to recruit activating proteins in a very transient way, which would not be captured by our purification and mass spectrometric analysis.

### Dynamic gene regulation and slower turnover of chromatin marks

Both the replacement of Cbx7-containing PRC1 for Cbx8 loaded complexes and the complete eviction of all PRC1 after prolonged activation occurs in the context of persisting H3K27me3 and H2A ubiquitination. This reiterates that these marks have a slow turnover and are not necessary a reflection of gene activity or repression, in particular during dynamic cell fate transitions. On Cbx8 target genes we could detect low levels of H3K27ac that were further reduced mildly but significantly in cells depleted for Cbx8 (S5B Figure). Whether Cbx8-dependent H3K27ac is a cause or consequence of enhanced transcription is unclear. H3K27ac could positively affect chromatin accessibility for transcription-supporting factors. However it is more likely that the observed low level of H3K27ac is a collateral consequence of an increased concentration of histone acetylases travelling with Polymerase II.

### PRC1 modularity during ES cell differentiation

The modularity of PRC1 is illustrated by the fact that there are 180 theoretical combinations for assembling the different PRC subunits. The real number of different PRC1 complexes existing under different physiological conditions is likely to be much lower due to mutually exclusive expression patterns and preferential binding between subunits. The systematic proteomic and epigenomic analysis of all six PCGF proteins shed some initial light on the different compositions and genomic distributions of PRC1 complexes [28]. On the one hand proteomic studies allowed the identification of new PRC1 components such as RYBP and YAF2, while on the other hand they increased doubts about the genuineness of subunits that had previously been considered to be canonical; such as Cbx6. A major challenge for the field is to understand how the modularity of PRC1 is regulated and how it contributes to cellular functions. Self-renewing ES cells primarily express two PRC1 complexes containing either Cbx7 or RYBP [7]. Whereas Cbx7-PRC1 mediates early repression of differentiation genes by binding to H3K27me3, RYBP-PRC1 binds independently of the methylation status of H3K27 and is associated with lower levels of Ring1b and H2A ubiquitination and occupies genes that are less repressed [29]. Cbx7 and Cbx8 are expressed in an almost mutually exclusive manner in self-renewing and differentiating ES cells, respectively. Although *in vitro* the chromodomain of Cbx7 has higher affinity to H3K27me3 than the one of Cbx8 [30], in cells we found that both proteins were able to efficiently replace each other on genes. Enforced expression of Cbx7 in differentiating cells was able to compete with Cbx8 for its target genes and to reduce gene activation. In the converse experiment similarly efficient replacement of Cbx7 by exogenous Cbx8 in self-renewing ES cells was not sufficient to induce derepression (Fig. 8A–D). These results place Cbx7 over Cbx8 in the functional hierarchy of Polycomb proteins. Future studies will have to assess in greater detail how different PRC1 complexes regulate cell fate decisions. Our preliminary data shows that ES cells that maintain 30–50% reduced Cbx8 expression are qualitatively able to differentiate into Tuj1-positive neurons with neurite outgrowth following 11 days of a long-term differentiation protocol (adapted from [31]), but seem to do so in a less efficient way (S6 Figure).

### Polycomb proteins and gene activation

The number of observations linking Polycomb proteins and active gene transcription are increasing. Here we report that the transient recruitment of PRC1 containing Cbx8 facilitates the transition from a Polycomb-repressed to a fully active state of key regulatory genes during early differentiation. Others have suggested that binding of Cbx8 to active genes could mark these for later repression [19]. Our data does not support this as we find Cbx8 recruitment to be only transient and all PRC1 to be entirely evicted after prolonged gene activation (Fig. 5).

It is worth to point out that Cbx8 target genes that got repressed after treatment with retinoic acid were not affected by the knockdown of Cbx8. This can be explained by a possible compensation by other canonical repressing PRC1 complexes or a recent finding showing that Polycomb protein recruitment is rather a consequence than a cause of initial gene silencing [32].

Studying differentiating myocytes, others have reported that Ezh1 associates with actively transcribed genes and further argued for a positive function in which Ezh1 could be required for the recruitment of Polymerase II [33]. In contrast, Pombo and colleagues suggested that their observed Polycomb-binding to transcribed metabolic genes was the consequence of a continuous switching between an active and a Polycomb-repressed state restraining transcriptional elongation [34]. In Drosophila cells, PRC1 was found to indirectly associate with some active as well as inactive genes by binding to structural cohesin proteins [35]. The authors argued again for a more active role on active genes by suggesting that PRC1 could be required for allowing the phosphorylation that makes Polymerase II elongation competent. A large body of additional work is needed to sort out the relation between Polycomb-bound and transcribed chromatin states in greater detail. In this endeavor it will be important to carefully distinguish between passive contributions from reductions in repressive potential and genuine contributions to transcription initiation and elongation.

## Materials and Methods

### Antibodies and plasmids

We produced a specific polyclonal antibody against mouse Cbx8 by immunizing rabbits with a His-tagged fragment of Cbx8 protein encompassing amino acids 201–360. Serum was precleared with sepharose and passed over a column containing a fusion protein of glutathione-S-transferase (GST) and amino acids 201–360 of Cbx8 covalently cross-linked to Glutathione sepharose. Anti-Cbx8 antibody was eluated with low pH, dialyzed and stored in PBS with 20% glycerol. The antibody performed in a similar way to antibodies previously described and kindly provided by Kristian Helin [13]; (S1 Figure). In addition we made use of the following antibodies: anti-IgG (Abcam), anti-H3 C-terminal (Abcam) and anti-H3K27me3 (Millipore), anti-Flag M2 (Sigma-Aldrich), anti-Ring1b provided by Luciano Di Croce [36], anti-H3K27Ac and anti-Cbx7 (Abcam), anti-H3K36me3 (Abcam) and rabbit monoclonal anti-ubiquityl-H2A (Lys119, D27C4, Cell Signaling Technology). The amounts and concentrations used for ChIP and western blotting is given in S3 Table. Expression plasmids and pLKO-1 constructs for shRNA-mediated knockdown were generated with standard PCR and cloning techniques. For stable expression in ES cells, cDNAs were cloned in frame with a multiple-epitope tag into a vector containing CAG promoter and an IRES-puromycin resistance gene cassette [37]. A modified and gateway cloning-adapted (Life Technologies) version was kindly provided by Diego Pasini.

### Cell culture, gene transduction and cell differentiation

E14Tg2A.4 mouse ES cells were cultured as previously described [38]. Cells were transduced with lentiviral shRNA cassettes essentially as described before [39]. Transduced cells were selected with 2 µg/ml puromycin. For shRNA sequences see S3 Table. For the generation of stably expressing ES cell clones, the above described CAG promoter driven vectors were transfected into mouse ES cells E14 using Lipofectamine 2000 (Life Technologies). Stable transfectants were selected with 2 µg/ml puromycin and analyzed by RT-PCR and western blot for transgene expression. For neuronal differentiation, 1 µM all trans retinoic acid (RA) was added directly to cells in medium without leukemia inhibitory factor. Unless indicated otherwise in Figure legends, cells were collected after 3 days of RA treatment.

### Protein and RNA analysis

Lysis and western blot analyses were performed as previously described [40]. Following the supplier's instructions, RNA was purified from 2×10^6^ cells using the RNeasy minikit (Qiagen), with a DNase I digestion step to avoid any potential DNA contamination. Total RNA (1 µg) was reverse transcribed using a cDNA synthesis kit (Roche Diagnostics) and oligo(dT) primers. Relative cDNA levels were quantified by quantitative PCR (qRT-PCR). Values were normalized to the expression of two housekeeping genes (*Rpo* and *Gapdh*). For gene expression analysis, four biological replicates of RA treated (3 days) and untreated ES cells were used for each condition and samples were prepared and hybridized to SurePrint G3 Mouse GE 8×60K Microarrays (Agilent technologies) following the supplier's instructions. Analyses were essentially performed as described [41] selecting differentially expressed probes with a FDR of 0.05 and fold change of >1.5.

### ChIP and ChIP-seq

Chromatin fragmented to a size ranging from 300–500 bps and immunoprecipitation (ChIP) experiments were performed essentially as previously described [42]. ChIP-reChIP experiment was performed as described elsewhere [34]. The sequences of all oligonucleotides used here are provided in the S3 Table. Unless indicated otherwise, ChIP results are given as the percentage of the amount of ChIP-enriched DNA relative to the amount of DNA isolated from one tenth of input material measured by quantitative PCR. For ChIP-sequencing (ChIP-seq), 10 ng of DNA was enriched by ChIP and fluorimetrically quantified with PicoGreen. Library generation and direct massive parallel sequencing on an Illumina genome analyzer were performed according to the supplier's instructions. Reads obtained were cleaned based on quality, trimmed using the ShortRead package in R [43] and aligned with the mouse genome (NCBIM37/mm9) using Bowtie version 0.12.7 [44], two mismatches were allowed for the alignment within the seed, only reads mapping to a single position in the genome were used. To detect genomic regions with significant enrichment we used MACS software version 1.4.1 [45]. For peak calling of Cbx8 in RA-treated ES cells we used a p-value cut-off of 1×10^−4^ and a FDR of 5%. Both IgG and Cbx8 from self-renewing cells (that do not express Cbx8) were independently used as control libraries. Only peaks called in both cases (minimal overlap of 50 bps) were accepted as high confidence target peaks and further analyzed. A subset was validated by direct ChIP. Peaks were annotated using ChIPpeakAnno package [46]. Genes were considered to be target genes if the center of a peak was found in the transcribed region ±3 kb using the transcript set of Mouse Ensembl Gene (based on assembly NCBIM37/mm9). In cases where a peak annotated to two genes, the nearest gene was selected and identified by the minimal distance between peak center and transcribed region. Un ambiguous peak that was found inside an intron of three differently annotated transcripts (chr10:3310058,3311329) was excluded from the analysis. To calculate the normalized enrichment profile we have used ngs.plot ver 2.0 [47]. The gene ontology analysis was performed using the function “getEnrichedGO” from ChippeakAnno package, we used the Ensembl gene ID and accepted GO categories with an adjusted P-value of 0.001 or less. P-values were calculated using the multiple adjusted Benjamini-Hochberg method. A value of ten was set as minimum count in the genome for a GO term to be included. For visualizing GO categories we used REVIGO [48], using the following parameters: “Medium” for the allowed similarity and “SimRel” for semantic similarity measure. Gene ontologies were grouped and genes were scored if they were associated to one or more ontology terms per group.

### Proteomic analysis

For proteomic analysis, 2×10^8^ differentiated ES cells expressing Flag-epitope tagged Cbx8 and parental control cells were collected with PBS. Nuclei were isolated using sucrose buffer and nuclear extract was separated from chromatinic fraction by a high salt extraction protocol followed by ultracentrifugation (1 h 50000 rpm). The soluble fraction containing nuclear extract was diluted to isotonic concentration followed by a preclear step using sepharose beads. Binding to Anti-Flag M2 Beads (Sigma-Aldrich) was performed in a rotating wheel during 2 h. Then, Anti-Flag M2 Beads were passed into a column for further enrichment and washing steps. Beads were collected into tubes for elution with 50 mM NaHCO_3_ and 0, 5% SDS. Eluated proteins were precipitated with cold acetone and frozen dry pellets were sent to a Proteomics facility for mass spectrometry analysis. Samples were digested with trypsin and 1 µg of each sample was injected in an Orbitrap Velos to LC-MS/MS analysis. Data was searched using an internal version of the search algorithm Mascot against a SwissProt_Mouse database (July 2013). Protein identification and peptides identified for each protein were identified using Proteome Discoverer v1.4, which gives an approximate estimation of protein amount with the average peak area of the 3 top peptides for a given protein. The number of peptides identified for each protein is a parameter of quality, the more peptides the better. Peptides were filtered based on the 1%FDR. Only proteins enriched more than 10 fold and with a coverage of at least 5% were considered for analysis.

### Statistics

Unless indicated otherwise, qRT-PCR and ChIP data is represented as the mean of three independent experiments and errors denote the standard deviation and stars indicate p-values below 0.05 as determined by the two-tailed Student's T-tests. Individual ChIP experiments are normalized to the average enrichment observed in the experiment. Means and errors of three experiments are scaled to represent the average of all experiments.

### Accession numbers

ChIP-seq and Microarray data have been deposited in the GEO database under accession number GSE54053.

## Supporting Information

S1 Figure Cbx8 induction during differentiation and generation of a specific Cbx8 antibody. (A) The relative mRNA levels of Cbx protein-encoding genes analyzed by qRT-PCR. Oct4 and Nanog were included as differentiation controls. Values from untreated cells were set to 1 and are plotted on a linear scale. (B) Schematic representation of the human CBX8 protein. Domain structure is according to Senthilkumar and Mishra [9]. The maximal sequence identity to other proteins is indicated for a 50 amino acid sliding window using the information provided by the human protein atlas project (www.proteinatlas.org). A stretch with minimal intraspecies identity to unrelated proteins and maximal conservation between mouse and human (>90% identity) was used as His-tagged antigen. (C) Scheme of the affinity purification of the antibody. Serum from immunized rabbits is precleared with sepharose and passed over a column containing a fusion protein of glutathione-S-transferase (GST) and amino acids 201–360 of CBX8 covalently cross (X)-linked to Glutathione sepharose. Anti-CBX8 antibody is eluated with low pH and stored in PBS with 20% glycerol. (D) Total cell lysates of NTera2/D1 (NT2) cells stably expressing shRNAs for CBX8 and controls were analyzed by western blot using the anti-CBX8 antibody. Anti- macroH2A1 antibody was used to control loading. (E) HEK293T cells were transiently transfected with HA-tagged CBX8 expression vector. Immunoprecipitations were performed under regular and chromatin immunoprecipitation (ChIP) conditions (crosslinking, stringent washing). As shown by western blot analysis using anti-HA antibody 5 µg anti-Cbx8 antibody performed well under both conditions. (F) Finally, we compared the performance of our anti-Cbx8 antibody (MB) to two well described antibodies that were kindly provided by Kristian Helin [13]. 5 µg of each antibody was used and IgG as control. As shown on 3 target genes in ES cells treated for three days with retinoic acid (RA), all three antibodies enriched similarly well. (G) Specificity of the ChIP signal is shown by the loss of signal in cells that have been infected with retroviral shRNA vectors specific for Cbx8 or controls.
(TIF)

S2 Figure Mapped reads and peak selection. Related to Fig. 1. (A) Details on the ChIP-seq data sets deposited in GEO. (B) Scheme for the identification of the subset of high confidence binding sites. We used MACS algorithm to identify peaks in the ES.Cbx8.RA library by comparing it independently to two control libraries, ES.IgG and ES.Cbx8.untreated. Overlapping peaks (min. 50 bp) are considered to represent 171 high confidence binding sites that have been analyzed further.
(TIF)

S3 Figure Gene ontologies and overlap with Ring1b and H3K27me3. (A) Bar graphs showing up to 30 top gene ontologies terms ordered for the –log_10_ adjusted p-value ≤0.001. Plots are shown for all genes annotated to Cbx8 peaks and also separately for up-, down- or not regulated genes considering a minimal fold-change of 1.2 comparing untreated and RA-treated cells. (B) Venn diagrams showing the overlap of Cbx8 target genes in RA-treated cells with those of Ring1b and H3K27me3 in untreated ES cells.
(TIF)

S4 Figure Influence of shRNA-mediated knockdown of Cbx8 or Ring1b on other PRC1 components and Cbx8 target genes. Related to Fig. 5. (A) FLAG-epitope tagged Cbx8 is detected in untreated ES cells binds to the same target genes as endogenous Cbx8 in RA-treated cells. ChIP data is shown. Error bars denote the variation of the mean of two independent experiments. (B) Knockdown of Cbx8 does not affect global Ring1b levels. Control and Cbx8-specific shRNA expressing cells were treated with RA for 3 days or left untreated. Lysates were analyzed by immunoblotting. (C) Repression of Cbx8 is not compensated by upregulation of Cbx2, Cbx4 or Cbx7. qRT-PCR analysis of mRNA levels in the same cells as A treated with RA for three days. Error bars denote s.d.; n = 3. (D) Control cells and cells expressing two different Ring1b-specific shRNAs were analyzed after three days of RA treatment. RNA was analyzed by qRT-PCR. Error bars denote s.d.; n = 3.
(TIF)

S5 Figure Low but detectable H3K27ac is sensitive to loss of Cbx8 in RA-treated ES cells. Related to Fig. 7. All experiments have been performed with ES cells treated with RA for three days, which is the time point of maximal Cbx8 recruitment (Fig. 5). (A) The H3K27ac observed by ChIP on Cbx8 target genes Sox9 and Tbx18 (average is plotted) is low compared to an enhancer with well known H3K27ac enrichment (Seq1 from [49]). (B) Histone H3K27ac levels on 4 Cbx8 target genes have been analyzed in control cells and in cells with shRNA-mediated knockdown of Cbx8. For each gene ChIP to input ratios of both are shown relative to the value from control cells expressing sh Scr. Error bars denote s.d.; n = 3; *, p<0.05. (compared to control cells).(C) Antibodies have been titrated by ChIP to precipitate similar levels of target gene DNA (left panel). Same amounts of antibodies were used to reChIP from anti-Cbx8 ChIP-enriched chromatin. Error bars represent the variation of the mean of two independent experiments.
(TIF)

S6 Figure Neuronal differentiation of ES cells with reduced Cbx8 expression. (A) Control and Cbx8 knockdown cells were plated onto bacteriological Greiner Petri dishes in 15 ml of EB medium in order to form embryoid bodies (EBs). Medium of EBs has been changed after 2 days. Another two days later, medium has been changed again and retinoic acid (RA) has been added. Another two days later, medium has been changed again with fresh medium containing RA. After 8 days of differentiation, the EBs have been dissociated using trypsin and plated in N2B27 medium onto polyornithine and laminin-coated plates. RNA was extracted at day 11 of the differentiation protocol and analyzed by qRT-PCR. Data is the mean of values obtained for two independent hairpins and is presented in respect to the value from control cells that has been set to 1. Error bars indicate the range of the individual values. (B) The same cells as in A were analyzed by immunofluorescence using antibody against the neuronal marker Tuj1.
(TIF)

S1 Table ChIP-seq peaks, annotated genes and their expression change after RA treatment. The table contains the data that was used to generate the Fig. 1D–F.
(XLSX)

S2 Table Proteomics data. Cbx8 and interacting proteins were enriched by affinity purification and analyzed by mass spectrometry and significantly enriched proteins were ranked according to their abundance. The table contains the list of results from which the table in Fig. 4B has been extracted.
(PDF)

S3 Table Oligo sequences and antibodies. The sequences of all oligos and their position is given. For antibodies we detail sources and amounts used for ChIP, IP and immunoblotting, respectively.
(XLSX)
