# Supplementary figures and images for: A Cbx8-Containing Polycomb Complex Facilitates the Transition to Gene Activation during ES Cell Differentiation
Source: PLoS Genet. 2014 Dec 11;10(12):e1004851. doi: 10.1371/journal.pgen.1004851 (PMC4263398; doi:10.1371/journal.pgen.1004851)

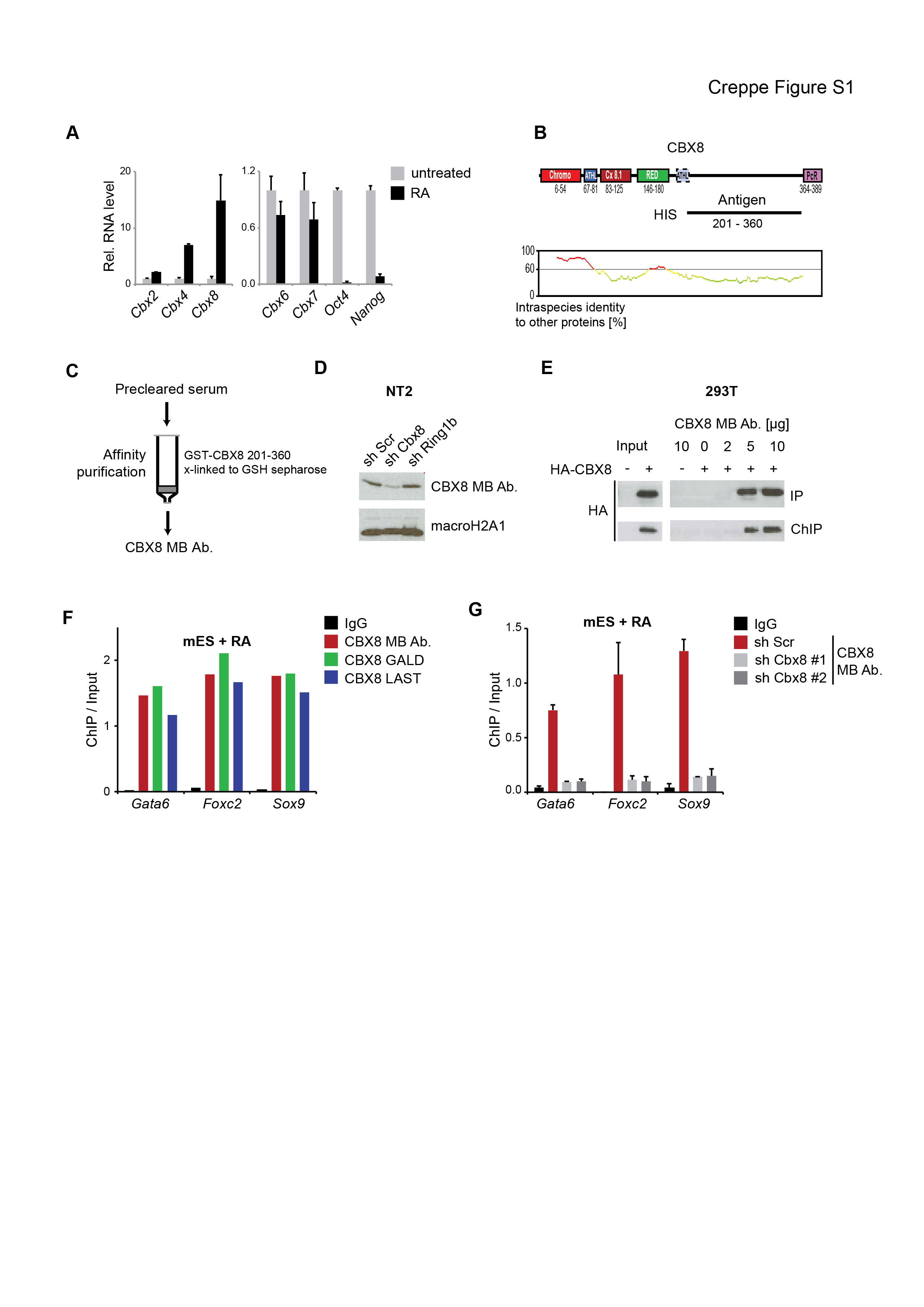

Supplement: S1 Figure — Cbx8 induction during differentiation and generation of a specific Cbx8 antibody. (A) The relative mRNA levels of Cbx protein-encoding genes analyzed by qRT-PCR. Oct4 and Nanog were included as differentiation controls. Values from untreated cells were set to 1 and are plotted on a linear scale. (B) Schematic representation of the human CBX8 protein. Domain structure is according to Senthilkumar and Mishra [9]. The maximal sequence identity to other proteins is indicated for a 50 amino acid sliding window using the information provided by the human protein atlas project (www.proteinatlas.org). A stretch with minimal intraspecies identity to unrelated proteins and maximal conservation between mouse and human (>90% identity) was used as His-tagged antigen. (C) Scheme of the affinity purification of the antibody. Serum from immunized rabbits is precleared with sepharose and passed over a column containing a fusion protein of glutathione-S-transferase (GST) and amino acids 201–360 of CBX8 covalently cross (X)-linked to Glutathione sepharose. Anti-CBX8 antibody is eluated with low pH and stored in PBS with 20% glycerol. (D) Total cell lysates of NTera2/D1 (NT2) cells stably expressing shRNAs for CBX8 and controls were analyzed by western blot using the anti-CBX8 antibody. Anti- macroH2A1 antibody was used to control loading. (E) HEK293T cells were transiently transfected with HA-tagged CBX8 expression vector. Immunoprecipitations were performed under regular and chromatin immunoprecipitation (ChIP) conditions (crosslinking, stringent washing). As shown by western blot analysis using anti-HA antibody 5 µg anti-Cbx8 antibody performed well under both conditions. (F) Finally, we compared the performance of our anti-Cbx8 antibody (MB) to two well described antibodies that were kindly provided by Kristian Helin [13]. 5 µg of each antibody was used and IgG as control. As shown on 3 target genes in ES cells treated for three days with retinoic acid (RA), all three antibodies en [file pgen.1004851.s001.tif]

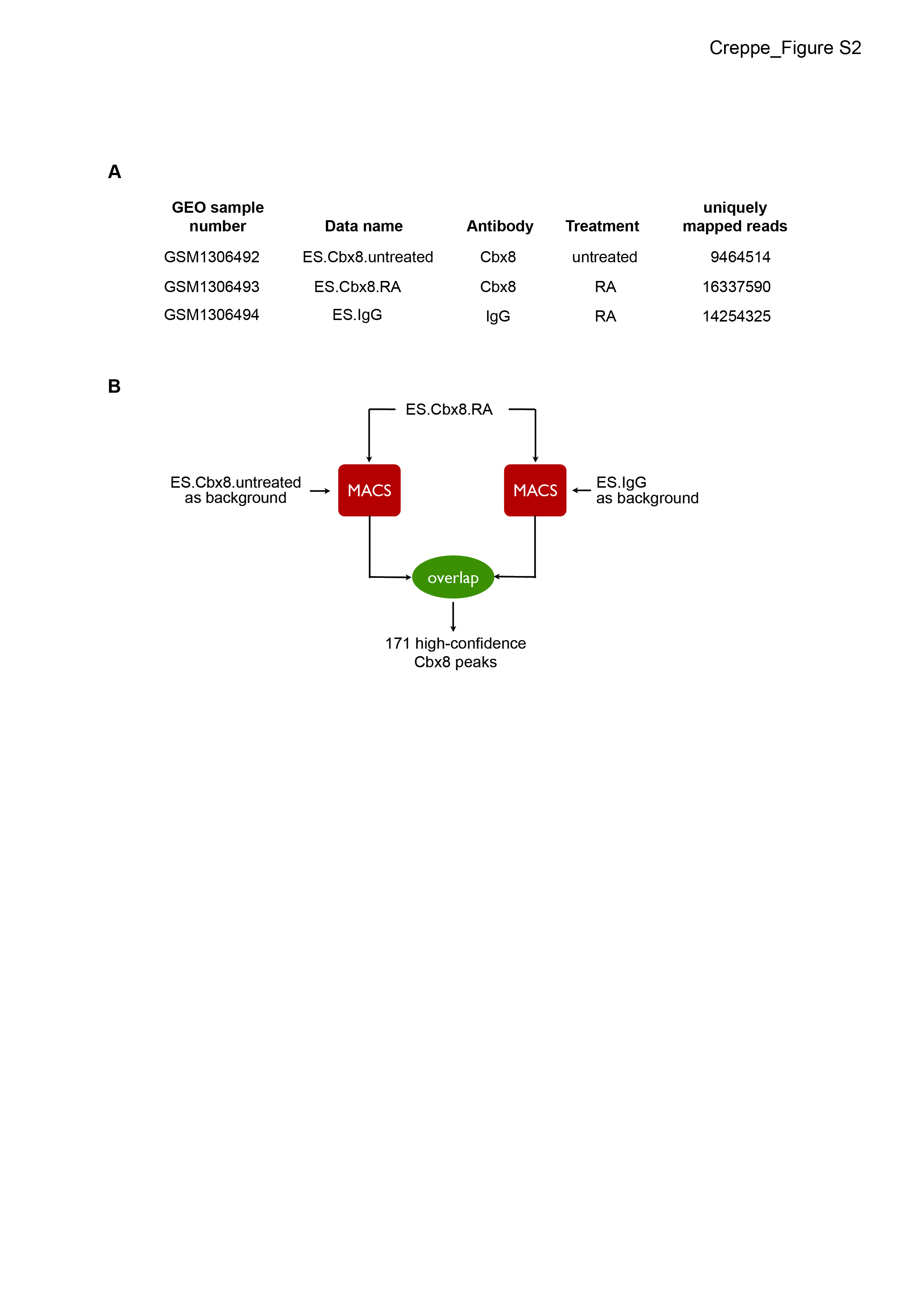

Supplement: S2 Figure — Mapped reads and peak selection. Related to Fig. 1. (A) Details on the ChIP-seq data sets deposited in GEO. (B) Scheme for the identification of the subset of high confidence binding sites. We used MACS algorithm to identify peaks in the ES.Cbx8.RA library by comparing it independently to two control libraries, ES.IgG and ES.Cbx8.untreated. Overlapping peaks (min. 50 bp) are considered to represent 171 high confidence binding sites that have been analyzed further. (TIF) [file pgen.1004851.s002.tif]

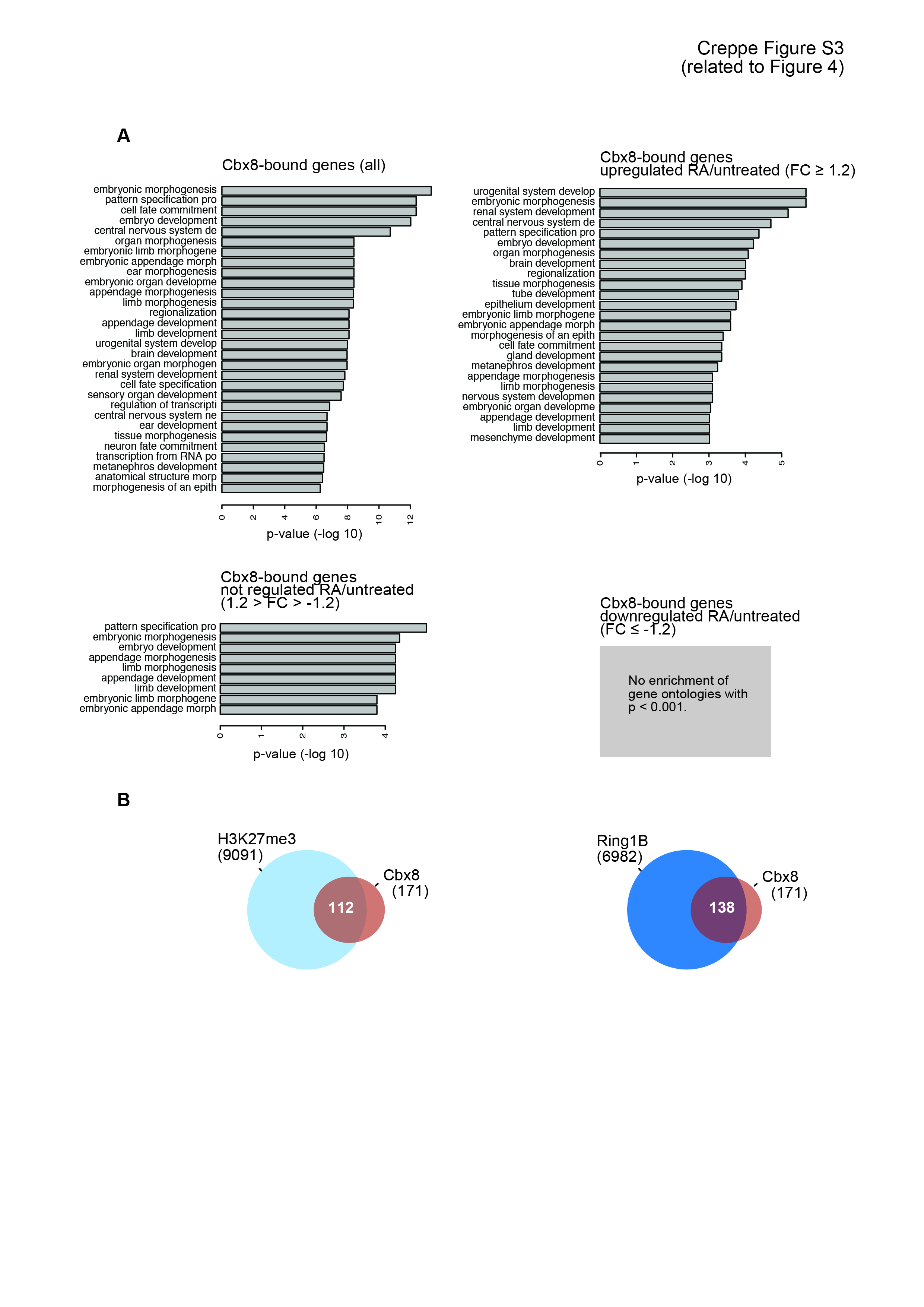

Supplement: S3 Figure — Gene ontologies and overlap with Ring1b and H3K27me3. (A) Bar graphs showing up to 30 top gene ontologies terms ordered for the –log10 adjusted p-value ≤0.001. Plots are shown for all genes annotated to Cbx8 peaks and also separately for up-, down- or not regulated genes considering a minimal fold-change of 1.2 comparing untreated and RA-treated cells. (B) Venn diagrams showing the overlap of Cbx8 target genes in RA-treated cells with those of Ring1b and H3K27me3 in untreated ES cells. (TIF) [file pgen.1004851.s003.tif]

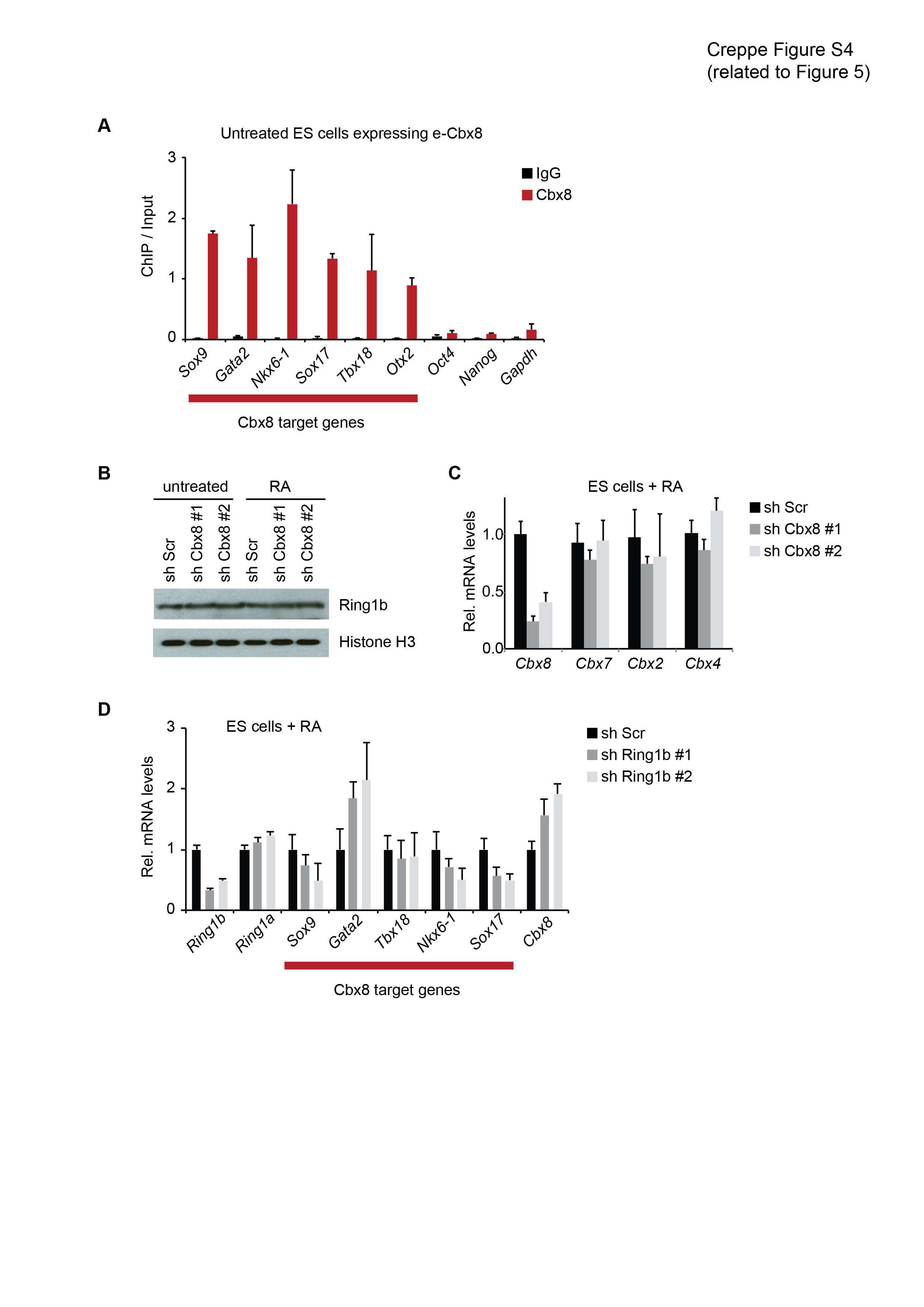

Supplement: S4 Figure — Influence of shRNA-mediated knockdown of Cbx8 or Ring1b on other PRC1 components and Cbx8 target genes. Related to Fig. 5. (A) FLAG-epitope tagged Cbx8 is detected in untreated ES cells binds to the same target genes as endogenous Cbx8 in RA-treated cells. ChIP data is shown. Error bars denote the variation of the mean of two independent experiments. (B) Knockdown of Cbx8 does not affect global Ring1b levels. Control and Cbx8-specific shRNA expressing cells were treated with RA for 3 days or left untreated. Lysates were analyzed by immunoblotting. (C) Repression of Cbx8 is not compensated by upregulation of Cbx2, Cbx4 or Cbx7. qRT-PCR analysis of mRNA levels in the same cells as A treated with RA for three days. Error bars denote s.d.; n = 3. (D) Control cells and cells expressing two different Ring1b-specific shRNAs were analyzed after three days of RA treatment. RNA was analyzed by qRT-PCR. Error bars denote s.d.; n = 3. (TIF) [file pgen.1004851.s004.tif]

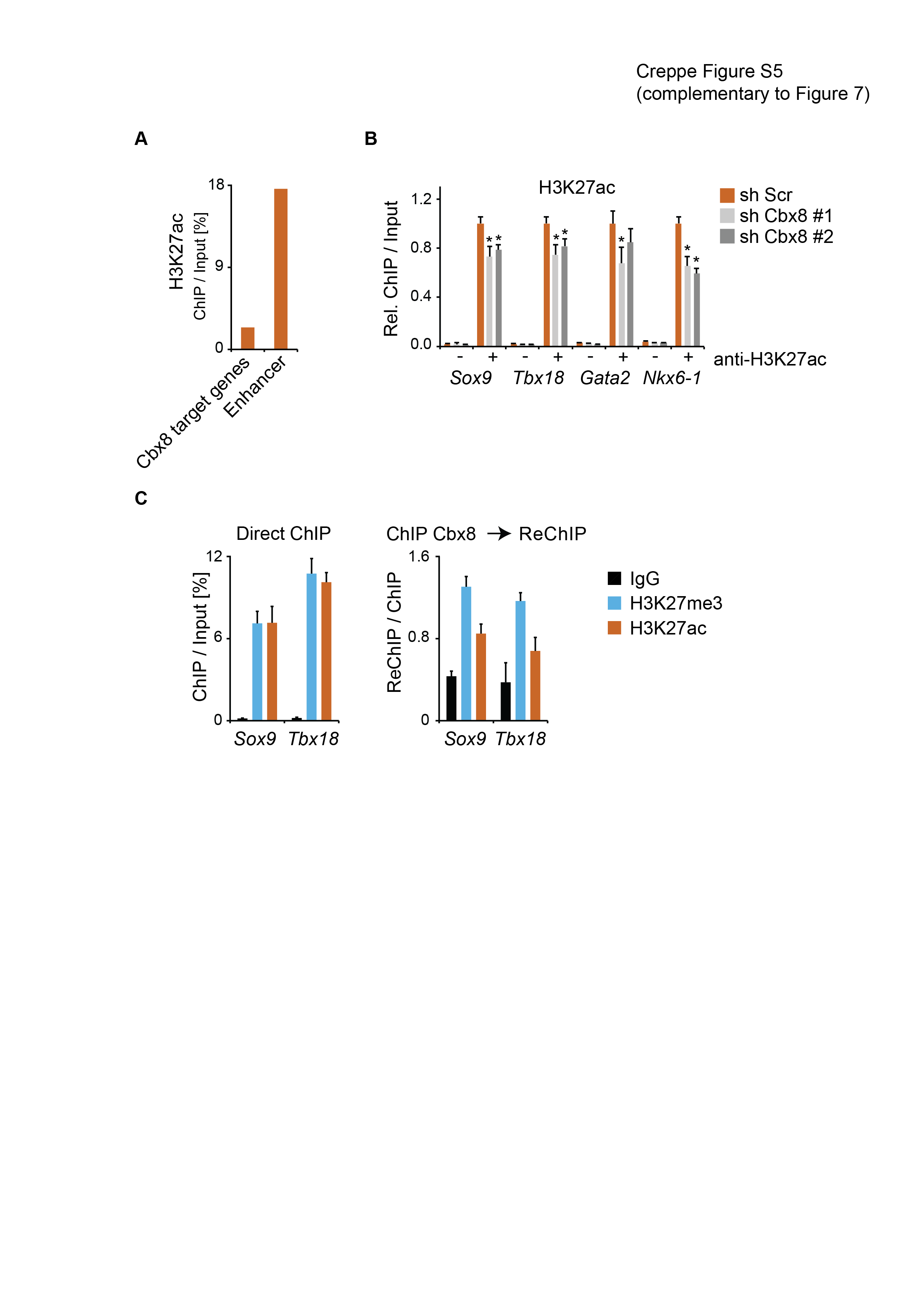

Supplement: S5 Figure — Low but detectable H3K27ac is sensitive to loss of Cbx8 in RA-treated ES cells. Related to Fig. 7. All experiments have been performed with ES cells treated with RA for three days, which is the time point of maximal Cbx8 recruitment (Fig. 5). (A) The H3K27ac observed by ChIP on Cbx8 target genes Sox9 and Tbx18 (average is plotted) is low compared to an enhancer with well known H3K27ac enrichment (Seq1 from [49]). (B) Histone H3K27ac levels on 4 Cbx8 target genes have been analyzed in control cells and in cells with shRNA-mediated knockdown of Cbx8. For each gene ChIP to input ratios of both are shown relative to the value from control cells expressing sh Scr. Error bars denote s.d.; n = 3; *, p<0.05. (compared to control cells).(C) Antibodies have been titrated by ChIP to precipitate similar levels of target gene DNA (left panel). Same amounts of antibodies were used to reChIP from anti-Cbx8 ChIP-enriched chromatin. Error bars represent the variation of the mean of two independent experiments. (TIF) [file pgen.1004851.s005.tif]

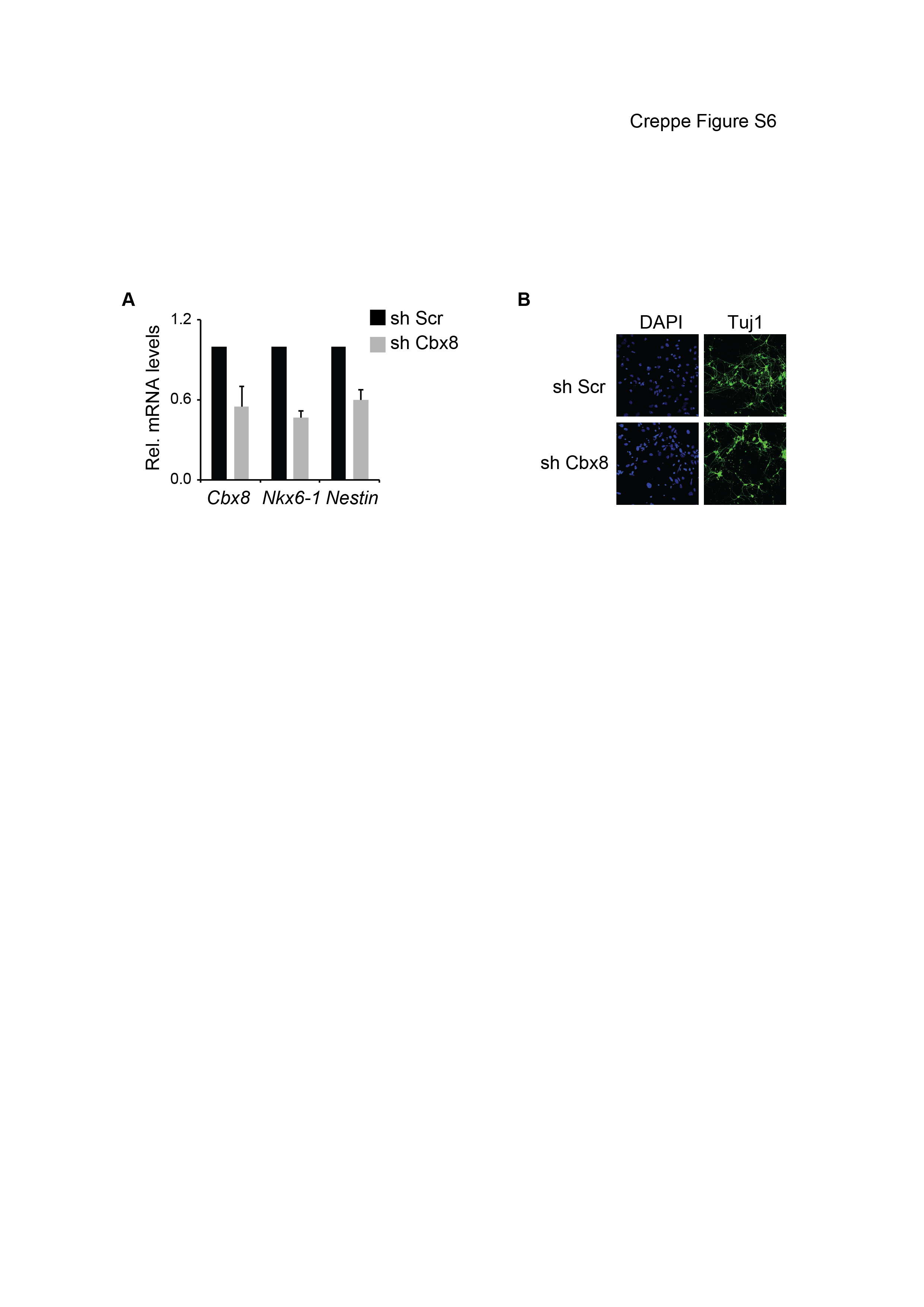

Supplement: S6 Figure — Neuronal differentiation of ES cells with reduced Cbx8 expression. (A) Control and Cbx8 knockdown cells were plated onto bacteriological Greiner Petri dishes in 15 ml of EB medium in order to form embryoid bodies (EBs). Medium of EBs has been changed after 2 days. Another two days later, medium has been changed again and retinoic acid (RA) has been added. Another two days later, medium has been changed again with fresh medium containing RA. After 8 days of differentiation, the EBs have been dissociated using trypsin and plated in N2B27 medium onto polyornithine and laminin-coated plates. RNA was extracted at day 11 of the differentiation protocol and analyzed by qRT-PCR. Data is the mean of values obtained for two independent hairpins and is presented in respect to the value from control cells that has been set to 1. Error bars indicate the range of the individual values. (B) The same cells as in A were analyzed by immunofluorescence using antibody against the neuronal marker Tuj1. (TIF) [file pgen.1004851.s006.tif]
