# Supplementary material for: A Cbx8-Containing Polycomb Complex Facilitates the Transition to Gene Activation during ES Cell Differentiation
Source: PLoS Genet. 2014 Dec 11;10(12):e1004851. doi: 10.1371/journal.pgen.1004851 (PMC4263398; doi:10.1371/journal.pgen.1004851)
Supplement: S2 Table — Proteomics data. Cbx8 and interacting proteins were enriched by affinity purification and analyzed by mass spectrometry and significantly enriched proteins were ranked according to their abundance. The table contains the list of results from which the table in Fig. 4B has been extracted. (PDF) [file pgen.1004851.s008.pdf]

Description	ΣCoverage		Σ# Unique Pe	Σ# Peptides	Σ# PSMs	Control	Flag-Cbx8			
Description	ΣCoverage	Σ# Proteins	Σ# Unique Peptide	Σ# Peptides	Σ# PSMs	A2: Area	B2: Area	ratio Area	Abundance relative to bait	Rank
CBX8 BAIT	50.55	1	19	19	116	0.000E0	7.719E9	100	100	1
RING2	39.58	2	12	14	42	3.884E7	3.585E9	92.30	46	2
RING1	23.15	1	5	7	12	4.557E7	1.775E9	38.96	23	3
PHC2	14.12	1	12	13	25	1.000E0	1.331E9	100	17	4
PCGF2 = MEL18	9.94	1	2	5	6	1.000E0	1.145E9	100	15	5
BMI1	8.02	1	1	4	5	1.000E0	8.942E8	100	9	6
60S ribosomal protein L10a OS=Mus musculus GN=Rpl10a PE=1 SV=3 - [RL10A_MOUSE]	29.03	1	7	7	10	5.557E7	7.325E8	13.18	9	7
Ig kappa chain V region Mem5 (Fragment) OS=Mus musculus PE=1 SV=1 - [KVM5_MOUSE]	5.79	1	1	1	1	1.000E0	7.226E8	100	9	8
ATP-dependent zinc metalloprotease YME1L1 OS=Mus musculus GN=Yme1l1 PE=2 SV=1 - [YMEL1_MOUSE]	5.73	1	2	2	3	1.000E0	6.839E8	100	9	9
PHC1	25.40	1	13	13	20	1.000E0	6.786E8	100	5	10
Cysteine and glycine-rich protein 2 OS=Mus musculus GN=Csrp2 PE=1 SV=3 - [CSRP2_MOUSE]	7.77	1	1	1	2	1.000E0	3.883E8	100	3	11
Serine/arginine-rich splicing factor 9 OS=Mus musculus GN=Srsf9 PE=1 SV=1 - [SRSF9_MOUSE]	16.67	1	3	4	5	1.000E0	2.121E8	100	2	12
S-phase kinase-associated protein 1 OS=Mus musculus GN=Skp1 PE=1 SV=3 - [SKP1_MOUSE]	17.79	1	1	1	7	8.216E6	1.925E8	23.43	2	13
Actin-related protein 2/3 complex subunit 5 OS=Mus musculus GN=Arpc5 PE=2 SV=3 - [ARPC5_MOUSE]	7.95	1	1	1	1	1.000E0	1.637E8	100	2	14
PCGF1 = NSPC1	15.44	1	2	2	2	1.000E0	1.564E8	100	2	15
PCGF6 = RNF134	24.08	1	5	5	8	1.000E0	1.564E8	100	2	16
Uncharacterized protein C7orf50 homolog OS=Mus musculus PE=2 SV=3 - [CG050_MOUSE]	12.31	1	1	1	1	1.000E0	1.505E8	100	2	17
MAX gene-associated protein OS=Mus musculus GN=Mga PE=2 SV=1 - [MGAP_MOUSE]	8.62	1	14	14	19	1.000E0	1.480E8	100	2	18
Pre-mRNA branch site protein p14 OS=Mus musculus GN=Sf3b14 PE=2 SV=1 - [PM14_MOUSE]	9.60	1	1	1	1	1.000E0	1.430E8	100	2	19
60S ribosomal protein L31 OS=Mus musculus GN=Rpl31 PE=2 SV=1 - [RL31_MOUSE]	34.40	1	4	4	5	1.000E0	1.360E8	100	1	20
Ribosomal RNA-processing protein 7 homolog A OS=Mus musculus GN=Rrp7a PE=2 SV=1 - [RRP7A_MOUSE]	22.50	1	3	3	4	1.000E0	1.322E8	100	1	21
40S ribosomal protein S13 OS=Mus musculus GN=Rps13 PE=1 SV=2 - [RS13_MOUSE]	16.56	1	3	3	4	1.000E0	1.223E8	100	1	22
RuvB-like 1 OS=Mus musculus GN=Ruvbl1 PE=1 SV=1 - [RUVB1_MOUSE]	19.74	1	5	5	6	1.000E0	1.062E8	100	1	23
40S ribosomal protein S15 OS=Mus musculus GN=Rps15 PE=2 SV=2 - [RS15_MOUSE]	26.90	1	2	2	2	1.000E0	9.890E7	100	1	24
Non-histone chromosomal protein HMG-17 OS=Mus musculus GN=Hmgn2 PE=1 SV=2 - [HMGN2_MOUSE]	16.67	1	1	1	1	1.000E0	9.543E7	100	1	25
U5 small nuclear ribonucleoprotein 40 kDa protein OS=Mus musculus GN=Snrnp40 PE=2 SV=1 - [SNR40_MOUSE]	17.60	1	2	2	3	1.000E0	9.475E7	100	1	26
DnaJ homolog subfamily A member 2 OS=Mus musculus GN=Dnaja2 PE=1 SV=1 - [DNJA2_MOUSE]	7.77	1	1	1	2	1.000E0	9.261E7	100	1	27
Neuroguidin OS=Mus musculus GN=Ngdn PE=1 SV=1 - [NGDN_MOUSE]	14.92	1	3	3	4	1.000E0	8.783E7	100	1	28
40S ribosomal protein S27 OS=Mus musculus GN=Rps27 PE=1 SV=3 - [RS27_MOUSE]	30.95	2	2	2	2	1.000E0	8.663E7	100	1	29
Protein virilizer homolog OS=Mus musculus GN=Kiaa1429 PE=1 SV=1 - [VIR_MOUSE]	5.80	1	7	7	8	6.384E6	8.387E7	13.14	1	30
Polycomb group RING finger protein 5 OS=Mus musculus GN=Pcgf5 PE=2 SV=1 - [PCGF5_MOUSE]	11.33	1	2	2	2	1.000E0	7.995E7	100	1	31
Splicing factor 1 OS=Mus musculus GN=Sf1 PE=1 SV=6 - [SF01_MOUSE]	7.81	1	2	2	4	1.000E0	7.670E7	100	1	32
Translocon-associated protein subunit delta OS=Mus musculus GN=Ssr4 PE=2 SV=1 - [SSRD_MOUSE]	6.40	1	1	1	1	1.000E0	7.639E7	100	1	33
Solute carrier family 2, facilitated glucose transporter member 3 OS=Mus musculus GN=Slc2a3 PE=1 SV=1 - [GTR3_MOUSE]	5.48	1	2	2	2	1.000E0	7.077E7	100	1	34
DnaJ homolog subfamily A member 3, mitochondrial OS=Mus musculus GN=Dnaja3 PE=1 SV=1 - [DNJA3_MOUSE]	9.38	1	3	3	4	1.000E0	6.974E7	100	1	35
60S ribosome subunit biogenesis protein NIP7 homolog OS=Mus musculus GN=Nip7 PE=2 SV=1 - [NIP7_MOUSE]	17.22	1	2	2	2	1.000E0	6.898E7	100	1	36
Mitochondrial 2-oxoglutarate/malate carrier protein OS=Mus musculus GN=Slc25a11 PE=1 SV=3 - [M2OM_MOUSE]	5.10	1	1	1	1	1.000E0	6.857E7	100	1	37
T-complex protein 1 subunit theta OS=Mus musculus GN=Cct8 PE=1 SV=3 - [TCPQ_MOUSE]	5.66	1	3	3	3	1.000E0	6.168E7	100	1	38
Origin recognition complex subunit 3 OS=Mus musculus GN=Orc3 PE=1 SV=1 - [ORC3_MOUSE]	10.63	1	5	5	6	1.000E0	6.038E7	100	1	39
Charged multivesicular body protein 4b OS=Mus musculus GN=Chmp4b PE=2 SV=2 - [CHM4B_MOUSE]	10.71	1	2	2	2	1.000E0	5.850E7	100	1	40
Protein max OS=Mus musculus GN=Max PE=1 SV=1 - [MAX_MOUSE]	6.25	1	1	1	1	1.000E0	5.796E7	100	1	41
Sequestosome-1 OS=Mus musculus GN=Sqstm1 PE=1 SV=1 - [SQSTM_MOUSE]	5.88	1	2	2	2	1.000E0	5.631E7	100	0	
Serpin H1 OS=Mus musculus GN=Serpinh1 PE=1 SV=3 - [SERPH_MOUSE]	9.35	1	3	3	3	1.000E0	5.546E7	100	0	
Coiled-coil-helix-coiled-coil-helix domain-containing protein 2, mitochondrial OS=Mus musculus GN=Chchd2 PE=1 SV=1 - [CH	15.69	1	1	1	1	1.000E0	5.467E7	100	0	
U2 small nuclear ribonucleoprotein A' OS=Mus musculus GN=Snrpa1 PE=1 SV=2 - [RU2A_MOUSE]	15.29	1	2	2	2	1.000E0	5.422E7	100	0	
Origin recognition complex subunit 4 OS=Mus musculus GN=Orc4 PE=1 SV=2 - [ORC4_MOUSE]	6.93	2	2	2	2	1.000E0	5.377E7	100	0	
Protein S100-A11 OS=Mus musculus GN=S100a11 PE=2 SV=1 - [S10AB_MOUSE]	26.53	1	2	2	2	1.000E0	5.240E7	100	0	
Cleavage and polyadenylation specificity factor subunit 4 OS=Mus musculus GN=Cpsf4 PE=2 SV=1 - [CPSF4_MOUSE]	14.69	1	2	2	2	1.000E0	5.191E7	100	0	
DNA-directed RNA polymerases I, II, and III subunit RPABC4 OS=Mus musculus GN=Polr2k PE=2 SV=2 - [RPAB4_MOUSE]	12.07	1	1	1	1	1.000E0	5.187E7	100	0	
T-complex protein 1 subunit eta OS=Mus musculus GN=Cct7 PE=1 SV=1 - [TCPH_MOUSE]	8.46	1	3	3	3	1.000E0	5.179E7	100	0	
Activating signal cointegrator 1 complex subunit 2 OS=Mus musculus GN=Ascc2 PE=2 SV=1 - [ASCC2_MOUSE]	5.21	1	2	2	2	1.000E0	5.166E7	100	0	
Cleavage stimulation factor subunit 2 OS=Mus musculus GN=Cstf2 PE=1 SV=2 - [CSTF2_MOUSE]	7.07	2	2	2	2	1.000E0	5.105E7	100	0	
Tubulointerstitial nephritis antigen-like OS=Mus musculus GN=Tinagl1 PE=1 SV=1 - [TINAL_MOUSE]	6.87	1	3	3	3	1.000E0	5.099E7	100	0	
Transcription factor E2F6 OS=Mus musculus GN=E2f6 PE=1 SV=2 - [E2F6_MOUSE]	6.25	1	1	1	2	1.000E0	4.839E7	100	0	
Nuclear envelope pore membrane protein POM 121 OS=Mus musculus GN=Pom121 PE=1 SV=2 - [PO121_MOUSE]	7.08	1	3	3	4	1.000E0	4.803E7	100	0	
Nucleoporin SEH1 OS=Mus musculus GN=Seh1l PE=2 SV=1 - [SEH1_MOUSE]	6.11	1	1	1	1	1.000E0	4.713E7	100	0	
Cell growth-regulating nucleolar protein OS=Mus musculus GN=Lyar PE=1 SV=2 - [LYAR_MOUSE]	5.15	4	2	2	2	1.000E0	4.664E7	100	0	

Chromatin complexes subunit BAP18 OS=Mus musculus GN=Bap18 PE=1 SV=1 - [BAP18_MOUSE]	8.19	1	1	1	1	1.000E0	4.481E7	100	0
ATP-binding cassette sub-family F member 2 OS=Mus musculus GN=Abcf2 PE=2 SV=1 - [ABCF2_MOUSE]	7.96	1	4	4	4	1.000E0	4.453E7	100	0
Small ubiquitin-related modifier 1 OS=Mus musculus GN=Sumo1 PE=1 SV=1 - [SUMO1_MOUSE]	21.78	1	1	1	1	1.000E0	4.222E7	100	
PAX3- and PAX7-binding protein 1 OS=Mus musculus GN=Paxbp1 PE=1 SV=3 - [PAXB1_MOUSE]	5.33	1	3	3	3	3.564E6	4.180E7	11.73	
Centrin-3 OS=Mus musculus GN=Cetn3 PE=2 SV=1 - [CETN3_MOUSE]	7.19	1	1	1	1	1.000E0	4.034E7	100	
Eukaryotic translation initiation factor 3 subunit F OS=Mus musculus GN=Eif3f PE=1 SV=2 - [EIF3F_MOUSE]	12.47	1	3	3	4	1.000E0	3.961E7	100	
Single-stranded DNA-binding protein, mitochondrial OS=Mus musculus GN=Ssbp1 PE=2 SV=1 - [SSBP_MOUSE]	5.26	1	1	1	1	1.000E0	3.842E7	100	
Protein LLP homolog OS=Mus musculus GN=Llph PE=2 SV=1 - [LLPH_MOUSE]	16.92	1	1	1	1	1.000E0	3.685E7	100	
40S ribosomal protein S17 OS=Mus musculus GN=Rps17 PE=1 SV=2 - [RS17_MOUSE]	23.70	1	2	2	4	1.000E0	3.635E7	100	
60S ribosomal protein L37a OS=Mus musculus GN=Rpl37a PE=2 SV=2 - [RL37A_MOUSE]	36.96	1	4	4	11	1.000E0	3.615E7	100	
Lupus La protein homolog OS=Mus musculus GN=Ssb PE=2 SV=1 - [LA_MOUSE]	9.40	1	3	3	3	1.000E0	3.556E7	100	
Myosin light chain 3 OS=Mus musculus GN=Myl3 PE=1 SV=4 - [MYL3_MOUSE]	14.22	1	2	2	2	1.000E0	3.497E7	100	
Dolichol-phosphate mannosyltransferase OS=Mus musculus GN=Dpm1 PE=2 SV=1 - [DPM1_MOUSE]	6.54	1	2	2	2	1.000E0	3.463E7	100	
Poly(rC)-binding protein 2 OS=Mus musculus GN=Pcbp2 PE=1 SV=1 - [PCBP2_MOUSE]	14.36	2	2	3	3	1.000E0	3.441E7	100	
Ragulator complex protein LAMTOR1 OS=Mus musculus GN=Lamtor1 PE=1 SV=1 - [LTOR1_MOUSE]	7.45	1	1	1	1	1.000E0	3.425E7	100	
Probable RNA-binding protein 19 OS=Mus musculus GN=Rbm19 PE=1 SV=1 - [RBM19_MOUSE]	5.57	1	3	3	3	1.000E0	3.379E7	100	
Ribonuclease P protein subunit p25-like protein OS=Mus musculus GN=Rpp25l PE=2 SV=1 - [RP25L_MOUSE]	5.52	1	1	1	1	1.000E0	3.368E7	100	
40S ribosomal protein S15a OS=Mus musculus GN=Rps15a PE=2 SV=2 - [RS15A_MOUSE]	6.15	1	1	1	1	1.000E0	3.323E7	100	
T-complex protein 1 subunit beta OS=Mus musculus GN=Cct2 PE=1 SV=4 - [TCPB_MOUSE]	13.83	1	5	5	6	1.000E0	3.301E7	100	
Serine/arginine-rich splicing factor 10 OS=Mus musculus GN=Srsf10 PE=1 SV=2 - [SRS10_MOUSE]	8.40	1	1	1	1	1.000E0	3.281E7	100	
Cytochrome P450 26A1 OS=Mus musculus GN=Cyp26a1 PE=2 SV=1 - [CP26A_MOUSE]	6.04	1	2	2	2	1.000E0	3.135E7	100	
DNA-directed RNA polymerase II subunit RPB11 OS=Mus musculus GN=Polr2j PE=2 SV=1 - [RPB11_MOUSE]	12.82	1	1	1	1	1.000E0	3.133E7	100	
Mitochondrial import inner membrane translocase subunit Tim23 OS=Mus musculus GN=Timm23 PE=2 SV=1 - [TIM23_MOUS	8.13	1	1	1	1	1.000E0	3.108E7	100	
Mesoderm-specific transcript protein OS=Mus musculus GN=Mest PE=2 SV=1 - [MEST_MOUSE]	11.04	1	2	2	2	1.000E0	3.079E7	100	
T-complex protein 1 subunit epsilon OS=Mus musculus GN=Cct5 PE=1 SV=1 - [TCPE_MOUSE]	5.36	1	2	2	2	1.000E0	3.021E7	100	
Chromobox protein homolog 5 OS=Mus musculus GN=Cbx5 PE=1 SV=1 - [CBX5_MOUSE]	6.81	1	1	1	1	1.000E0	2.893E7	100	
Mitochondrial ribonuclease P protein 1 OS=Mus musculus GN=Trmt10c PE=2 SV=2 - [MRRP1_MOUSE]	5.56	1	1	1	1	1.000E0	2.739E7	100	
DNA-directed RNA polymerase II subunit RPB4 OS=Mus musculus GN=Polr2d PE=2 SV=2 - [RPB4_MOUSE]	17.61	1	1	1	2	1.000E0	2.558E7	100	
Upstream-binding protein 1 OS=Mus musculus GN=Ubp1 PE=1 SV=1 - [UBIP1_MOUSE]	5.19	1	2	2	2	1.000E0	2.550E7	100	
mRNA turnover protein 4 homolog OS=Mus musculus GN=Mrto4 PE=2 SV=1 - [MRT4_MOUSE]	6.28	1	1	1	1	1.000E0	2.407E7	100	
Eukaryotic translation initiation factor 4E type 2 OS=Mus musculus GN=Eif4e2 PE=1 SV=1 - [IF4E2_MOUSE]	8.98	1	1	1	1	1.000E0	2.372E7	100	
Centrin-2 OS=Mus musculus GN=Cetn2 PE=1 SV=1 - [CETN2_MOUSE]	7.56	1	1	1	1	1.000E0	1.770E7	100	
Chromatin modification-related protein MEAF6 OS=Mus musculus GN=Meaf6 PE=2 SV=1 - [EAF6_MOUSE]	6.81	1	1	1	1	1.000E0	1.730E7	100	
26S proteasome non-ATPase regulatory subunit 14 OS=Mus musculus GN=Psmd14 PE=1 SV=2 - [PSDE_MOUSE]	10.97	1	1	1	1	1.000E0	1.556E7	100	
RNA-binding protein 7 OS=Mus musculus GN=Rbm7 PE=1 SV=1 - [RBM7_MOUSE]	6.79	1	1	1	2	1.000E0	1.507E7	100	
Homeobox protein Meis1 OS=Mus musculus GN=Meis1 PE=1 SV=1 - [MEIS1_MOUSE]	6.15	2	1	1	2	1.000E0	1.404E7	100	
Surfeit locus protein 4 OS=Mus musculus GN=Surf4 PE=2 SV=1 - [SURF4_MOUSE]	6.69	1	1	1	2	1.000E0	1.369E7	100	
28S ribosomal protein S6, mitochondrial OS=Mus musculus GN=Mrps6 PE=2 SV=3 - [RT06_MOUSE]	8.00	1	1	1	1	1.000E0	1.271E7	100	
Copine-1 OS=Mus musculus GN=Cpne1 PE=1 SV=1 - [CPNE1_MOUSE]	5.41	1	2	2	3	1.000E0	1.222E7	100	
Nucleolar protein 16 OS=Mus musculus GN=Nop16 PE=2 SV=1 - [NOP16_MOUSE]	8.99	1	1	1	1	1.000E0	8.285E6	100	
Ig kappa chain V-V region MOPC 149 OS=Mus musculus PE=1 SV=1 - [KV5A4_MOUSE]	12.04	1	1	1	1	1.000E0	7.270E6	100	
Protein SEC13 homolog OS=Mus musculus GN=Sec13 PE=2 SV=3 - [SEC13_MOUSE]	8.07	1	1	1	2	1.000E0	7.123E6	100	
Cyclin-dependent kinase 2-associated protein 1 OS=Mus musculus GN=Cdk2ap1 PE=1 SV=2 - [CDKA1_MOUSE]	11.40	1	1	1	1	1.000E0	3.253E6	100	

Contaminant

PRC1 component
